# Tumour Cell-Associated Heterogeneity in Antiviral Immunity Shapes Influenza Responses and Immune-Modulatory Signalling in Lung Cancer

**DOI:** 10.3390/cancers18213502

**Published:** 2026-10-30

**Authors:** Mark A. Miles, Isabella Rosano, Stella Liong, Jessica M. Logan, John J. O’Leary, Doug A. Brooks, Stavros Selemidis

**Affiliations:** 1 Centre for Respiratory Science and Health, School of Health and Biomedical Sciences, RMIT University, Bundoora, VIC 3083, Australia; isabella.rosano@rmit.edu.au (I.R.); stella.liong@rmit.edu.au (S.L.); 2 School of Pharmacy and Biomedical Science, College of Health, Adelaide University, North Terrace, Adelaide, SA 5001, Australia; jessica.logan@adelaide.edu.au (J.M.L.); douglas.brooks@adelaide.edu.au (D.A.B.); 3 Discipline of Histopathology, School of Medicine, Trinity Translational Medicine Institute (TTMI), Trinity College Dublin, XW7X Dublin, Ireland; olearyjj@tcd.ie; 4 Sir Patrick Dun’s Laboratory, Central Pathology Laboratory, St James’s Hospital, XW7X Dublin, Ireland; 5 CERVIVA Research Consortium, Trinity College Dublin, XW7X Dublin, Ireland

**Keywords:** lung cancer, influenza A virus, innate immune sensing, PD-L1, antiviral immunity

## Abstract

Background/Objectives: Respiratory viral infections occur within the same anatomical niche as lung cancer, yet how lung cancer cells respond to infection and how these responses influence antiviral and inflammatory signalling remain poorly understood. This study investigated innate immune responses to influenza A virus (IAV) infection across a panel of lung epithelial cell lines. Methods: Non-malignant bronchial epithelial and non-small cell lung cancer (NSCLC) cell lines representing adenocarcinoma and squamous cell carcinoma were infected with IAV. Antiviral and inflammatory gene expression, viral RNA and protein detection, infection-induced cell death, pattern recognition receptor expression, and immune checkpoint ligand expression were assessed using molecular, biochemical and flow cytometric approaches. Interferon (IFN) receptor blockade and mitochondrial reactive oxygen species scavenging were used to investigate selected signalling mechanisms. Results: NSCLC cell lines exhibited heterogeneous innate immune responses to IAV infection, with differential regulation of RNA-sensing pathways and canonical antiviral signalling components. Although IFN responses were retained, NSCLC cells exhibited reduced infection-induced apoptosis and prolonged viral RNA and protein detection compared with non-malignant epithelial cells. IAV infection was also associated with changes in DNA-sensing receptor expression, including TLR9, and increased expression of the immune checkpoint ligand PD-L1. Conclusions: These findings identify substantial cell line-associated heterogeneity in antiviral responses to IAV across lung epithelial cell lines, including NSCLC cells. The observed differences in viral RNA and protein detection, cell death responses and immune-modulatory signalling provide a framework for understanding how respiratory viral infections may interact with lung cancer cells to shape antiviral and inflammatory responses within the tumour environment.

## Simple Summary

Respiratory viruses infect the lungs, where they may also encounter lung cancer cells. However, how lung cancer cells directly respond to these infections remains poorly understood. This study investigated how different lung cancer cell lines respond to influenza A virus, focusing on antiviral defences, inflammation, cell death and immune-regulatory signals. We found substantial differences between cell lines in their antiviral responses, with lung cancer cells showing reduced infection-induced cell death, prolonged detection of viral material and changes in inflammatory and immune-regulatory pathways. These findings highlight the diversity of responses to respiratory viral infection across lung cancer cell models and provide a foundation for further research into how viral infections may interact with lung cancer biology and influence the tumour environment.

## 1. Introduction

Lung cancer remains the leading cause of cancer-related mortality worldwide, with non-small cell lung cancer (NSCLC) accounting for approximately 85% of cases [1]. NSCLC arises from epithelial cells lining the respiratory tract, positioning these tumour cells within the primary site of respiratory viral infection. Malignant epithelial cells are therefore not only exposed to pathogens, but may also serve as opportunistic targets for viral infection. While respiratory viral infections are well recognised to influence tumour progression by specific effects on the immune microenvironment [2], the consequences of infection within tumour cells themselves remain poorly defined.

Influenza A virus (IAV) is a single-stranded RNA (ssRNA) virus that infects epithelial cells of the respiratory tract and is a major cause of seasonal and pandemic disease. Viral recognition within epithelial cells is mediated by pattern recognition receptors (PRRs), including endosomal toll-like receptors (TLR3 and TLR7) and cytosolic RNA sensors such as retinoic acid-inducible gene-I (RIG-I), which activate downstream signalling pathways leading to type I and III interferon (IFN) production and induction of antiviral effector responses [3]. These pathways are critical for limiting viral replication and promoting infection-related cell death, thereby restricting the spread of the virus. However, many of these same signalling networks are dysregulated in NSCLC. For instance, TLR signalling in lung cancer exhibits dual function [4]. Activation within immune cells can enhance anti-tumour immunity through dendritic cell, natural killer cell and T cell activation [5]. On the other hand, tumour-intrinsic TLR signalling has been associated with increased survival, chemoresistance and metastatic potential, partly through NF-κB activation and upregulation of anti-apoptotic proteins such as Bcl-2 [6]. TLR7, in particular, is upregulated in some lung tumours and correlates with aggressive disease and poor clinical outcomes [7,8]. Such alterations in PRR expression, IFN signalling, and downstream transcriptional pathways are common features of malignant transformation and are associated with tumour cell survival, immune evasion and therapeutic resistance [9]. These changes raise the possibility that antiviral responses in tumour cells may be functionally altered, not only affecting how viruses are sensed, but also how effectively the infection is controlled.

Emerging evidence suggests that respiratory viral infections can modulate lung tumour progression, with outcomes dependent on timing, tumour stage and immune context. Epidemiological studies suggest exposure to IAV may be associated with increased lung cancer risk, although causality remains unclear [10,11,12]. Experimental models further demonstrate that the effects of viral infection on tumour progression are context dependent. Intranasal IAV infection can exacerbate established NSCLC by promoting T cell exhaustion and enhancing programmed death-ligand 1 (PD-L1) expression on tumour cells, thereby contributing to an immunosuppressive microenvironment [13]. IAV infection before tumour establishment has similarly been associated with increased tumour burden and mortality via impairment of anti-tumour immune responses [14]. Comparable redirection of tumour-specific cytotoxic T cell responses following IAV infection has also been reported in melanoma and colorectal cancer models [15,16]. Moreover, IAV or severe acute respiratory syndrome coronavirus 2 infection can promote the expansion of dormant disseminated breast cancer cells in the lung by altering T cell responses, increasing metastatic burden [17]. Conversely, other models have demonstrated beneficial effects of prior IAV infection, including enhanced immune surveillance and reduced tumour burden [18,19]. In spontaneous NSCLC models, IAV infection has also been associated with macrophage repolarisation towards a more inflammatory, anti-tumour phenotype [20]. Collectively, these findings highlight the complex and context-dependent effects of respiratory viral infections on cancer progression. While much of the existing evidence has focused on virus-induced changes within the tumour microenvironment, particularly macrophage and T cell responses, considerably less is known about how malignant epithelial cells themselves recognise and respond to respiratory viral infection. Specifically, whether tumour cell-intrinsic differences in antiviral sensing and signalling influence viral dynamics, cell survival and downstream immune-modulatory responses remains poorly understood.

We hypothesised that lung cancer cells exhibit distinct tumour cell-intrinsic antiviral responses to IAV infection. This may potentially create an intracellular environment with differing capacities for viral control and downstream immune signalling. To address this, we investigated cytokine responses, viral dynamics, PRR expression and immune checkpoint expression in a panel of NSCLC cell lines following IAV infection. In doing so, we sought to characterise how cell line-associated differences in antiviral sensing relate to viral burden, cell death responses and downstream immune signalling pathways.

## 2. Materials and Methods

### 2.1. Cell Lines

Non-malignant bronchial epithelial cells (BEAS-2B), lung adenocarcinoma cell lines (A549, CALU-3), and lung squamous cell carcinoma cell lines (H520, SK-MES-1) were used in this study. A549 (RRID:CVCL_0023) and BEAS-2B (RRID:CVCL_0168) cells were cultured in Advanced Dulbecco’s Modified Eagle Medium (DMEM)/F-12 (Invitrogen, Carlsbad, CA, USA), while CALU-3 (RRID:CVCL_0609), H520 (RRID:CVCL_1566) and SK-MES-1 (RRID:CVCL_0630) cells were cultured in Roswell Park Memorial Institute (RPMI) + GlutaMax media (Invitrogen). All culture medium was supplemented with 10% (*v*/*v*) foetal bovine serum (FBS; Scientifix, Melbourne, Australia) and 1% (*v*/*v*) penicillin–streptomycin (Invitrogen). Cells were maintained at 37 °C in 5% CO_2_ and passaged by trypsinisation using TrypLE Express (Invitrogen). SK-MES-1 cells were purchased from Merck Life Science (Baywater, VIC, Australia) while all other cell lines were originally purchased from ATCC (Manassas, VA, USA).

### 2.2. Virus Infection and Agonist Treatments

The A/Puerto Rico/8/34 (PR8) H1N1 strain of IAV was used in this study. Original viral stocks were provided by Professor Patrick Reading (The Doherty Institute, Melbourne, Australia) and propagated in 10-day-old specific pathogen-free embryonated chicken eggs. Virus titres were quantified using standard plaque assay protocols in MDCK cells, with stock concentrations prepared at 7.5 × 10^7^ plaque-forming units/millilitre (PFU/mL), stored as single-use aliquots at −80 °C. The TLR7 agonist imiquimod (IMQ) and TLR3 agonist Polyinosinic:Polycytidylic Acid (Poly(I:C)) were purchased from InvivoGen (San Diego, CA, USA).

For viral and agonist exposure, cells were seeded in 6-well plates at a density of 1.5 x 10^5^ cells per well in the afternoon and allowed to adhere overnight. In initial experiments, an additional well was seeded in parallel for each cell line and harvested to determine the cell number at the time of inoculation. Cell numbers at inoculation were generally comparable to the initial seeding density; therefore, the initial seeding density was used to estimate cell number at inoculation in subsequent experiments and to calculate MOI accordingly. The following morning, the culture medium was replaced with a medium containing virus or agonist. Poly(I:C) and IMQ were delivered extracellularly to achieve final concentrations of 1 or 10 µg/ml. Virus- or agonist-containing medium was maintained for the duration of the experiment, with no post-adsorption wash performed. Cells were then harvested at specified timepoints (stated in figure legends) and processed for subsequent analyses. For infection analysis at days 3 or 7, cultures were replaced with fresh media after the initial 24 h in virus and passaged on days 3 or 5 until harvesting. In some experiments, cells were pretreated with 0.5 µg/mL human IFNAR1 antibody (Anti-hIFNAR-hIgG1-FES; Invivogen) or 50 µM mitoTEMPO (#SML0737; Sigma, Bayswater, VIC, Australia) for 1 h prior to infection. Corresponding control conditions consisted of cells receiving no blocking antibody or no inhibitor, respectively.

### 2.3. Propidium Iodide Uptake Assay

Virus-induced cell death was determined via flow cytometry using a propidium iodide (PI) uptake assay to measure cell membrane integrity. Following incubation with virus, media containing dead cells were collected and remaining attached cells were harvested by trypsinisation. Cell pellets containing viable and dead cells were resuspended in PBS containing 1 µg/mL PI (Invitrogen). Fluorescence intensity was quantified using the LSR Fortessa X-20 flow cytometric analyser (BD Biosciences, Franklin Lakes, NJ, USA) with a 561 nm laser and YG 610/20 emission filter by acquiring at least 10,000 total events. The representative gating strategy is shown in Supplementary Figure S1.

### 2.4. Caspase Activity Assay

Caspase-3/7 activity was measured using the Caspase-Glo 3/7 Assay System (Promega; Madison, WI, USA) and reported as DEVDase activity. Three thousand cells were seeded per well of white-walled flat 96-well plates. Culture medium containing the virus was added to a total volume of 60 µL and cells were incubated for specified times. Caspase-Glo 3/7 reagent (20 µL) was mixed with each well and plates were incubated for 30 min. Luminescence was read at 9 points per well using the Clariostar (BMG; Mornington, VIC, Australia).

### 2.5. Gene Expression Analysis

Total RNA was extracted using the RNAeasy Mini Kit (Qiagen; Germantown, MD, USA) with RNA concentration and purity determined using a Nanodrop One Spectrophotometer (Thermofisher Scientific, Scoresby, VIC, Australia). Five hundred nanograms of total RNA was reverse transcribed into copy DNA (cDNA) using the High-Capacity cDNA Reverse Transcription Kit (Thermofisher Scientific) according to the following settings: 25 °C for 10 min, 37 °C for 120 min, followed by 85 °C for 5 min. Quantitative polymerase chain reaction was carried out using the TaqMan Fast Advanced Master Mix (Life Technologies, Carlsbad, CA, USA) and analysed on the QuantStudio 7 Flex Real-Time PCR system (Life Technologies). The following TaqMan assay IDs (Life Technologies) were used in this study: *IL6* (Hs00174131_m1), *TNF* (Hs00174128_m1), *IFNB1* (Hs01077958_s1), *IFNL2/3* (Hs04193049_gH), *TLR3* (Hs01551079_g1), *TLR7* (Hs00152971_m1), *DDX58* (Hs01061436_m1), *TLR9* (Hs00152973_m1), *CGAS* (Hs00403553_m1), *ISG15* (Hs00192713_m1), *PDL1* (Hs00204257_m1), *PDL2* (Hs00228839_m1), and *18S* (Hs99999901_s1). Expression of influenza A polymerase gene was evaluated using a custom-designed TaqMan primer containing the oligonucleotide sequences 5′-CGGTCCAAATTCCTGCTGA-3′ and 5′-CATTGGGTTCCTTCCATCCA-3′. The following programme settings were used for amplification: 50 °C for 2 min, 95 °C for 2 min, then 40 cycles of 95 °C for 1 s and 60 °C for 20 s. The quantitative values were obtained from the average threshold cycle (Ct) number of each sample run in triplicate and gene expression analysis performed using the comparative Ct method. Target gene expression was normalised against 18S rRNA reference gene expression for each sample and expressed relative to the uninfected/untreated controls (2^−ΔΔCt^). Samples for which no amplification was detected were reported as undetermined by the instrument and assigned a Ct value of 40 for subsequent analysis. The stability of 18S rRNA expression across cell lines and PR8 infection conditions was assessed by examining raw 18S Ct values from the corresponding qPCR experiments (Supplementary Figure S2).

### 2.6. Western Blotting

Cells were lysed in RIPA buffer (150 mM sodium chloride, 1.0% Triton X-100, 0.5% sodium deoxycholate, 0.1%SDS, 50 mM Tris, pH 8.0) containing a protease inhibitor cocktail (Sigma) and supernatants cleared by centrifugation at 10,000× *g* for 15 min at 4 °C. Protein levels in lysates were quantitated using the Pierce BCA Protein Assay Kit (Thermofisher Scientific) according to the manufacturer’s instructions. Ten micrograms of protein was separated by SDS-PAGE, transferred to PVDF membranes (BioRad Laboratories, Hercules, CA, USA), and then membranes were blocked in 5% (*w*/*v*) skim milk for 1 h at room temperature. Transfer membranes were incubated in primary antibodies diluted in TBS-T buffer (Tris buffered saline containing 0.1% (*v*/*v*) Tween20), washed, and then probed with HRP-conjugated secondary antibodies diluted in TBS-T buffer. Protein bands were detected using the Western Lightning Ultra Chemiluminescent Substrate (Revvity; Scoresby, VIC, Australia). The following Cell Signalling Technologies (Danvers, MA, USA) antibodies were used unless otherwise stated: rabbit anti-TLR3 (#6961), rabbit anti-TLR7 (#5632), mouse anti-TLR9 (#AB134368; Abcam, Cambridge, Cambridgeshire, UK), rabbit anti-RIG-I (#3743), rabbit anti-MYD88 (#4283), rabbit anti-TRIF (#PA5-88150; Themofisher Scientific), rabbit anti-MAVS (#83000), mouse anti-Actin (#3700), rabbit anti-PARP (#9532), mouse anti-Influenza A NP (#AB128193; Abcam), horse anti-mouse-IgG-HRP (#7076), and goat anti-rabbit-IgG-HRP (#7074). Uncropped blots are shown in Supplementary Figure S3.

### 2.7. PD-L1 Cell Surface Staining

Following infection, cells were harvested and stained with PE/Cyanine5-conjugated anti-human PD-L1 (CD274/B7-H1; clone 29E.2A3; BioLegend, San Diego, CA, USA) or the corresponding PE/Cyanine5-conjugated mouse IgG2b, κ isotype control (clone MCP-11; BioLegend) at a 1:300 dilution in PBS containing 2% FBS. After incubation on ice for 30 min, cells were washed, resuspended in staining buffer, and analysed on a BD LSRFortessa™ X-20 flow cytometer (BD Biosciences). Cells were initially gated based on forward- and side-scatter characteristics to exclude debris and events with atypical scatter profiles, and mean fluorescence intensity (MFI) was determined using FlowJo software (Tree Star, Inc., Ashland, OR, USA). The representative gating strategy is shown in Supplementary Figure S1.

### 2.8. Cellular DNA Analysis by Microscopy

One hundred thousand cells were seeded on glass cover slips in 6-well plates a day prior to infection. Following infection, media was removed and cells fixed in 4% paraformaldehyde for 15 min. Cells were then stained with Quant-iT PicoGreen dsDNA Reagent (Thermofisher Scientific) diluted 1:200 in PBS for 30 min. Coverslips were then mounted on microscope slides with Fluoromount-G with DAPI (Thermofisher Scientific) and imaged using a Zeiss Axiovert 5 Digital microscope (Zeiss, Macquarie Park, NSW, Australia). Images were acquired from randomly selected, non-overlapping fields of view across the coverslip, avoiding areas with obvious cell aggregation or other imaging artefacts. The percentage of cells with punctate cytosolic PicoGreen staining was determined by counting 100 cells across randomly selected fields of view. Quantification was performed without blinding to experimental condition.

### 2.9. Statistical Analysis

Statistical analyses were performed using GraphPad Prism (version 10.1.2; GraphPad Software, San Diego, CA, USA). Data is reported as mean ± standard error of the mean (± SEM) from independent experiments with the number of biological replicates specified in the corresponding figure legends. Statistical significance was determined using one-way or two-way ANOVA followed by multiple comparisons tests where appropriate. Significance was considered with *p*-values below 0.05.

## 3. Results

### 3.1. IAV Infection Induces Variable Levels of Apoptotic Cell Death in NSCLC Cell Lines

**Figure 1:**
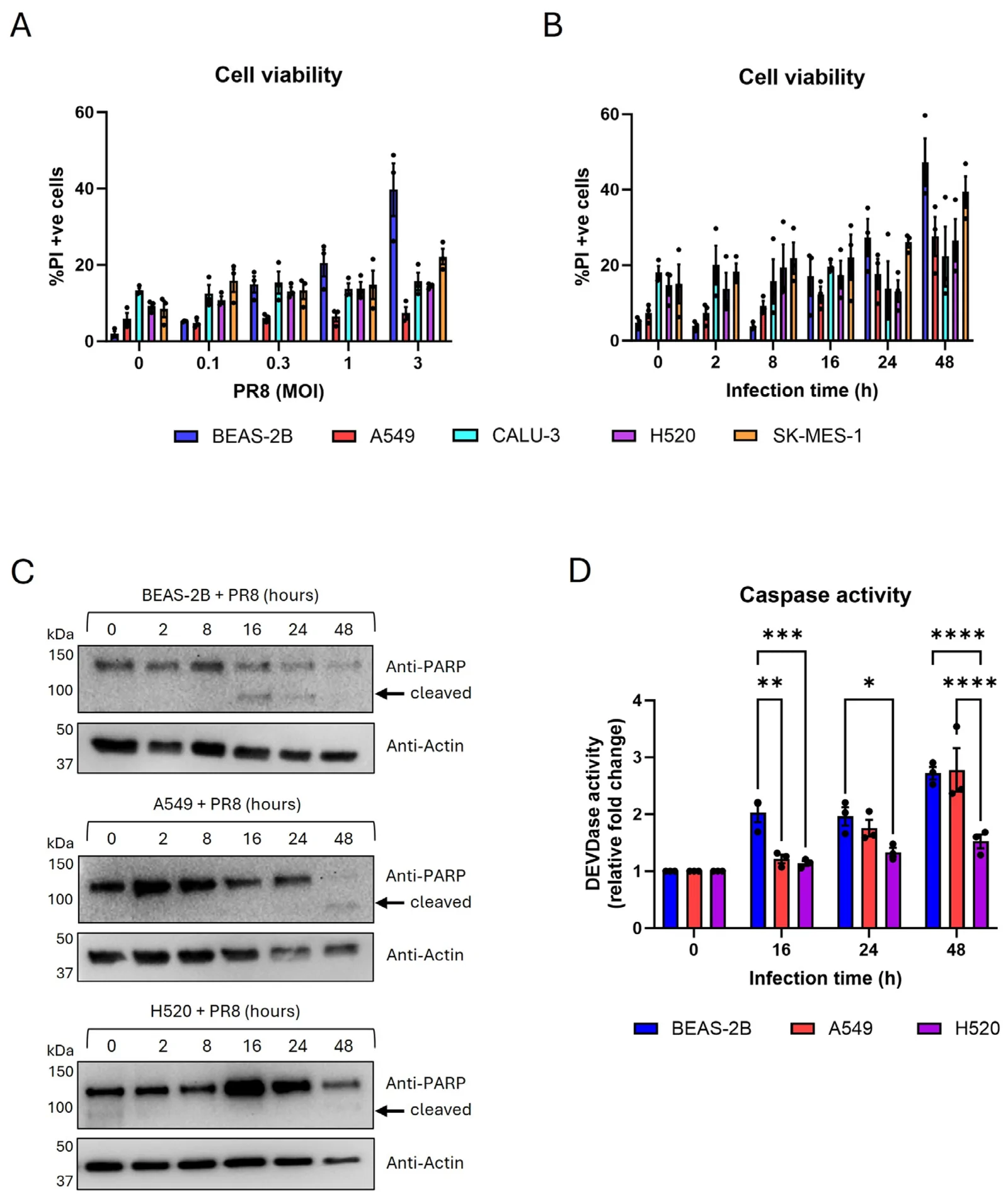
NSCLC cells fail to efficiently undergo IAV-induced apoptotic cell death. Malignant A549, CALU-3, H520 and SK-MES-1 and non-malignant BEAS-2B lung epithelial cell lines were infected with PR8 virus at (**A**) increasing multiplicity of infection (MOI) for 48 h or (**B**) MOI of 1 for various incubation times. The proportion of dead cells was measured by the percentage of propidium iodide (PI) stained cells. Additional experiments were performed in BEAS-2B, A549 and H520 cells infected with PR8 virus (MOI of 1) and analysed at various timepoints. (**C**) Western blotting was performed to probe for full-length and cleaved PARP expression, and (**D**) DEVDase activity was measured by Caspase-3/7 Glo assay and presented relative to uninfected cells. Data are expressed as mean ± SEM from 3 independent biological replicates. Western blot data are representative of a single experiment. Statistical analysis was conducted using a two-way ANOVA test followed by Tukey’s post hoc test for multiple comparisons (* *p* < 0.05, ** *p* < 0.01, *** *p* < 0.001, **** *p* < 0.0001). The original image of western blot are shown in Supplementary File S1.

To examine whether susceptibility to IAV-induced cytotoxicity differed across lung epithelial cell lines, we infected a panel of lung epithelial cell lines, representing non-malignant epithelium (BEAS-2B), adenocarcinoma (A549, CALU-3) and squamous cell carcinoma (H520, SK-MES-1), with increasing multiplicity of infection (MOI) of PR8 virus. Non-malignant BEAS-2B cells exhibited a dose- and time-dependent increase in cell death, with ~40% cell death at the highest dose (Figure 1A) and latest timepoint (Figure 1B). The malignant NSCLC cell lines A549, CALU-3 and H520 did not show an increase in PI +ve cells relative to their uninfected controls. SK-MES-1 cells showed a slight increase in cell death at the highest viral dose and latest timepoint. Consistent with this observation, cleaved PARP was detected in BEAS-2B cells from 16 h post-infection (Figure 1C) and coincided with increased DEVDase activity at this timepoint (Figure 1D), suggesting apoptotic caspases were active. In A549 cells, PARP cleavage was detected only at 48 h, while no cleavage was observed in H520 cells, consistent with delayed or absent DEVDase activity in these specific malignant cell lines.

Together, these findings indicate that the malignant lung epithelial cell lines examined exhibited minimal infection-associated apoptotic responses, unlike non-malignant BEAS-2B cells under the experimental conditions used.

### 3.2. NSCLC Cell Lines Exhibit Variable Antiviral Cytokine Responses Following IAV Infection

**Figure 2:**
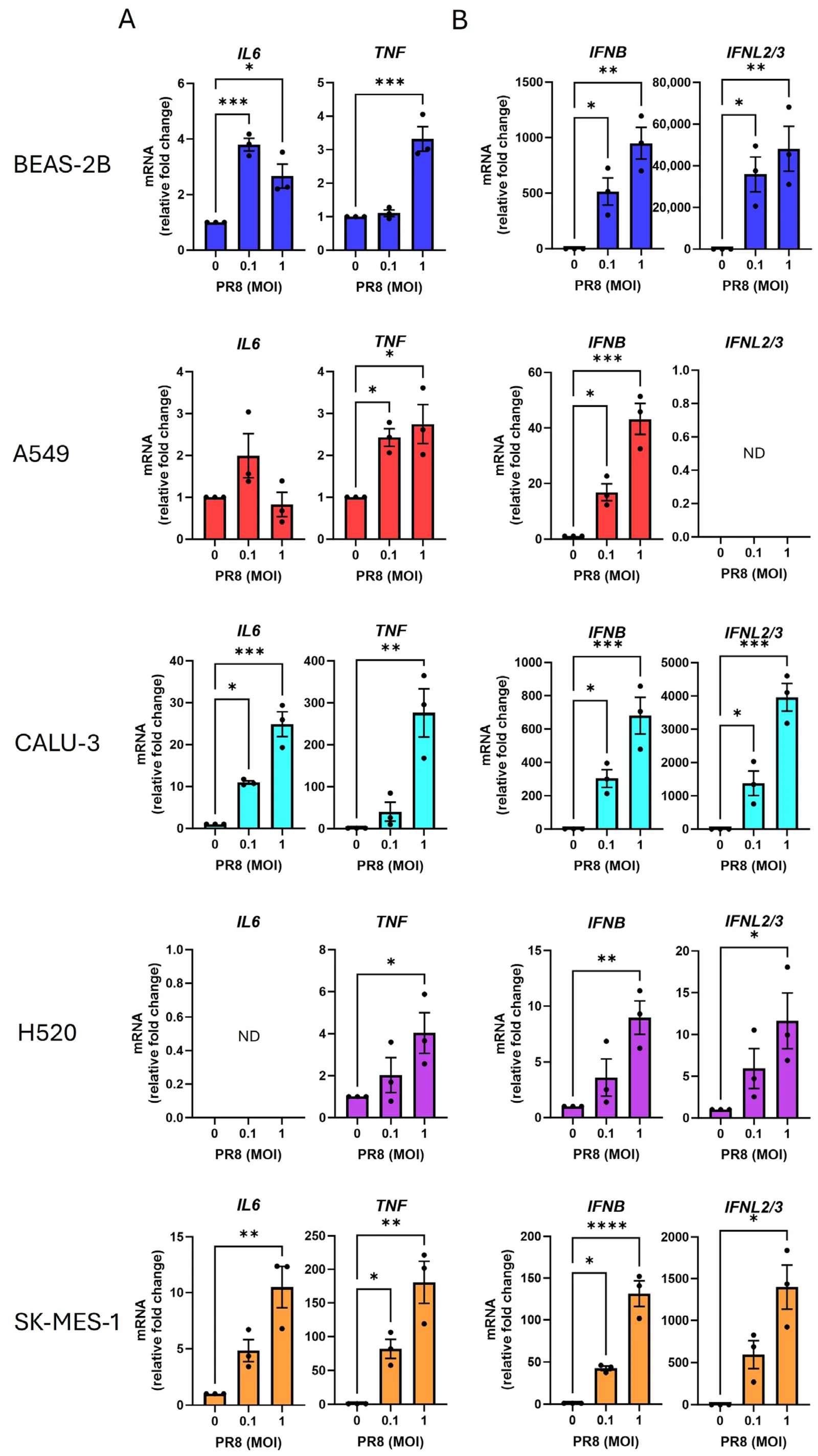
IAV infection induces variable cytokine expression in NSCLC cells. Malignant A549, CALU-3, H520 and SK-MES-1 and non-malignant BEAS-2B lung epithelial cell lines were infected with PR8 virus at MOI of 0, 0.1 and 1 for 24 h. Gene expression of (**A**) proinflammatory and (**B**) antiviral cytokines was measured by RT-qPCR. Data are presented relative to 18S housekeeping as a fold-change above uninfected controls (2^−ΔΔCt^). Genes that failed to reach cycle threshold across the sample group and were below the limit of quantification are denoted as “ND” (not detected). Data are expressed as mean ± SEM from 3 independent biological replicates. Statistical analysis was conducted using a one-way ANOVA test followed by Dunnett’s post hoc test for multiple comparisons (* *p* < 0.05, ** *p* < 0.01, *** *p* < 0.001, **** *p* < 0.0001).

Given the differences in apoptotic response to infection among the cell lines, we next examined proinflammatory and antiviral cytokine responses with viral dynamics following PR8 infection. *IL6* induction was observed in BEAS-2B, CALU-3 and SK-MES-1 cells but was undetectable in H520 cells (Figure 2A). *TNF* was significantly upregulated in all cell lines. Similarly, type I (*IFNB*) and type III (*IFNL2/3*) IFN induction also occurred in the majority of cells (Figure 2B). Notably, *IFNL2/3* was undetectable in A549 cells under both basal and infected conditions, indicating selective impairment of type III IFN production in this cell line.

**Figure 3:**
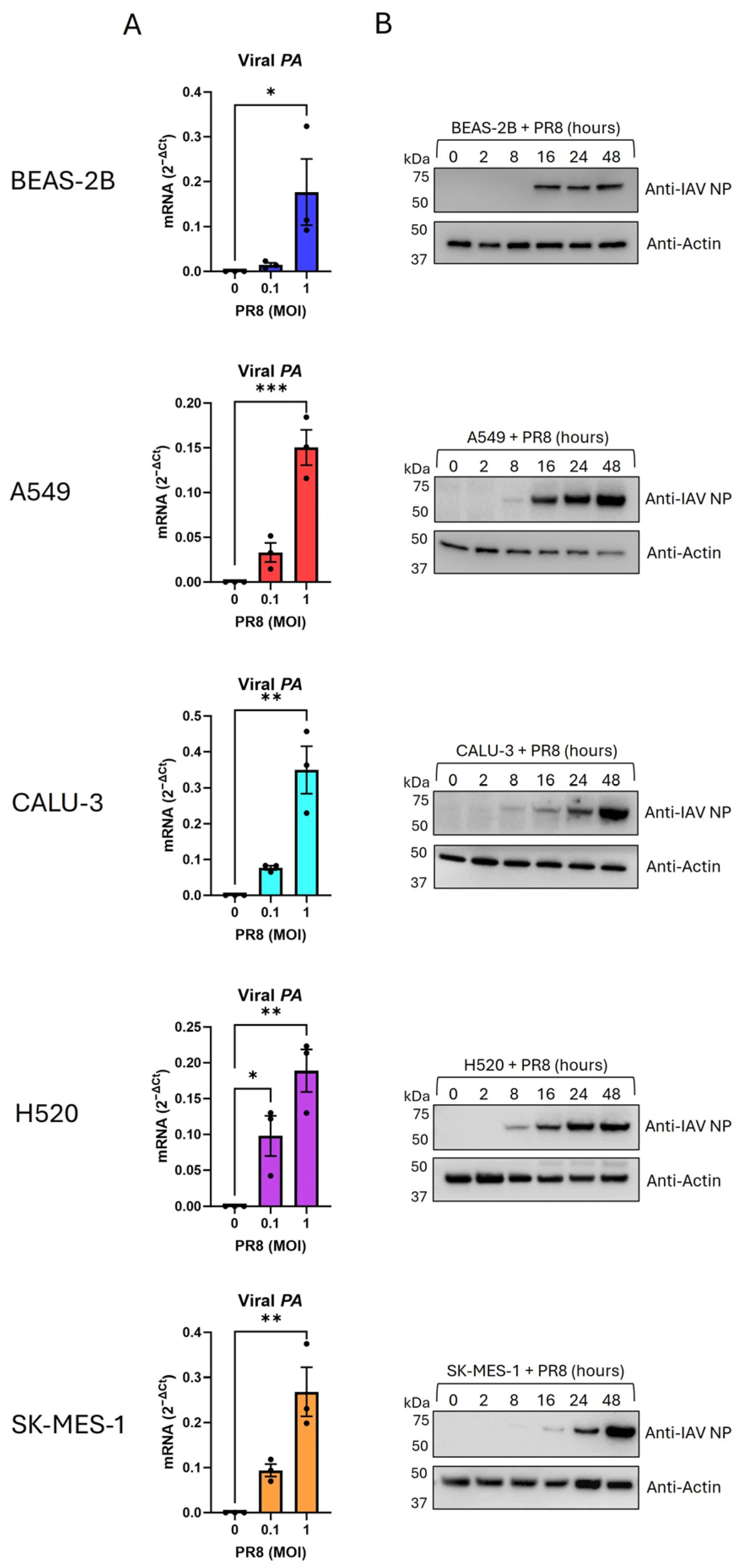
Accumulation of viral protein in lung epithelial cells following IAV infection. Malignant A549, CALU-3, H520 and SK-MES-1 and non-malignant BEAS-2B lung epithelial cell lines were infected with PR8 virus. (**A**) Following infection with MOI of 0, 0.1 and 1 for 24 h, expression of viral PA transcripts was measured by RT-qPCR and is presented relative to 18S housekeeping (2^−ΔCt^). Data are expressed as mean ± SEM from 3 independent biological replicates. (**B**) Cells were infected with PR8 virus (MOI of 1) and Western blotting for viral nucleoprotein (NP) was performed at various timepoints. Blots are representative of a single experiment. Statistical analysis was conducted using a one-way ANOVA test followed by Dunnett’s post hoc test for multiple comparisons (* *p* < 0.05, ** *p* < 0.01, *** *p* < 0.001). The original image of western blot are shown in Supplementary File S1.

**Figure 4:**
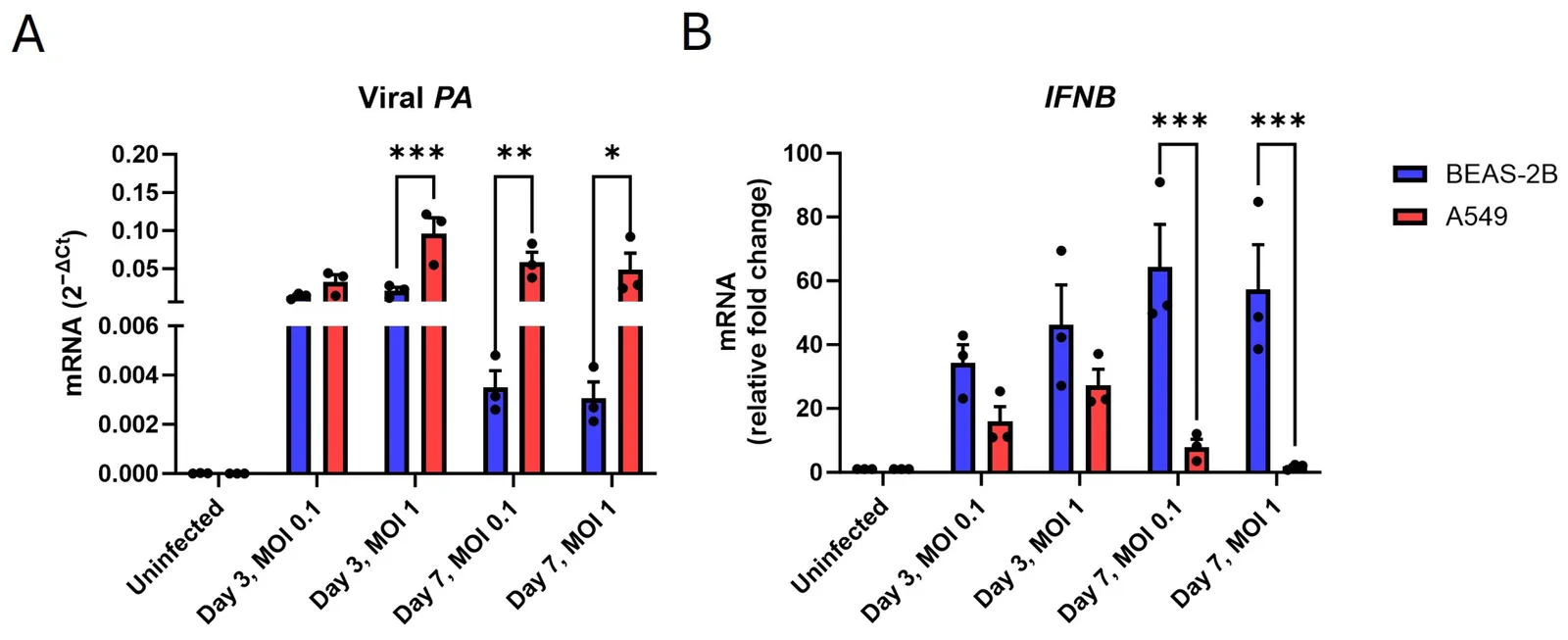
Higher viral transcripts are detected in malignant lung epithelial cells. Malignant A549 and non-malignant BEAS-2B lung epithelial cell lines were infected with PR8 virus and cultured for 3 or 7 days. RT-qPCR was performed to measure mRNA expression of (**A**) viral PA transcripts, presented relative to 18S housekeeping (2^−ΔCt^), or (**B**) IFNB, presented as a fold-change above uninfected controls (2^−ΔΔCt^). Data are expressed as mean ± SEM from 3 independent biological replicates. Statistical analysis was conducted using a two-way ANOVA test followed by Sidak’s post hoc test for multiple comparisons (* *p* < 0.05, ** *p* < 0.01, *** *p* < 0.001).

To determine whether these differences in cytokine gene expression responses reflected altered viral burden, we measured viral transcript and protein expression. Viral polymerase acidic protein transcripts (*PA*) were detected in all lines in a dose-dependent manner, confirming infection (Figure 3A). Nucleoprotein (NP) protein was detectable from 8 to 16 h onward in all of the epithelial cells, confirming accumulation of viral protein within 48 h following infection (Figure 3B). Based on the blots, NP protein expression in malignant cell lines appeared to increase over time whereas its levels beyond 16 h remained stable in BEAS-2B cells. This suggests the potential for a greater capacity for sustained accumulation of viral protein in the NSCLC cell lines. To examine whether viral RNA remained detectable at later timepoints, we measured *PA* transcripts in A549 and BEAS-2B cultures after 3 and 7 days following infection. Viral transcripts were detected in both cultures at these later timepoints, although significantly more transcripts were present in A549 compared to BEAS-2B cells (Figure 4A). Sustained *IFNB* upregulation was also detected at these timepoints with BEAS-2B cells exhibiting significantly higher levels (Figure 4B).

Collectively, these findings demonstrate that the non-malignant and NSCLC cell lines we tested induced distinct antiviral cytokine responses to IAV infection with an accumulation of viral protein observed in NSCLC cells.

### 3.3. NSCLC Cell Lines Display Heterogeneous Basal PRR Protein Expression and Signalling Responses

**Figure 5:**
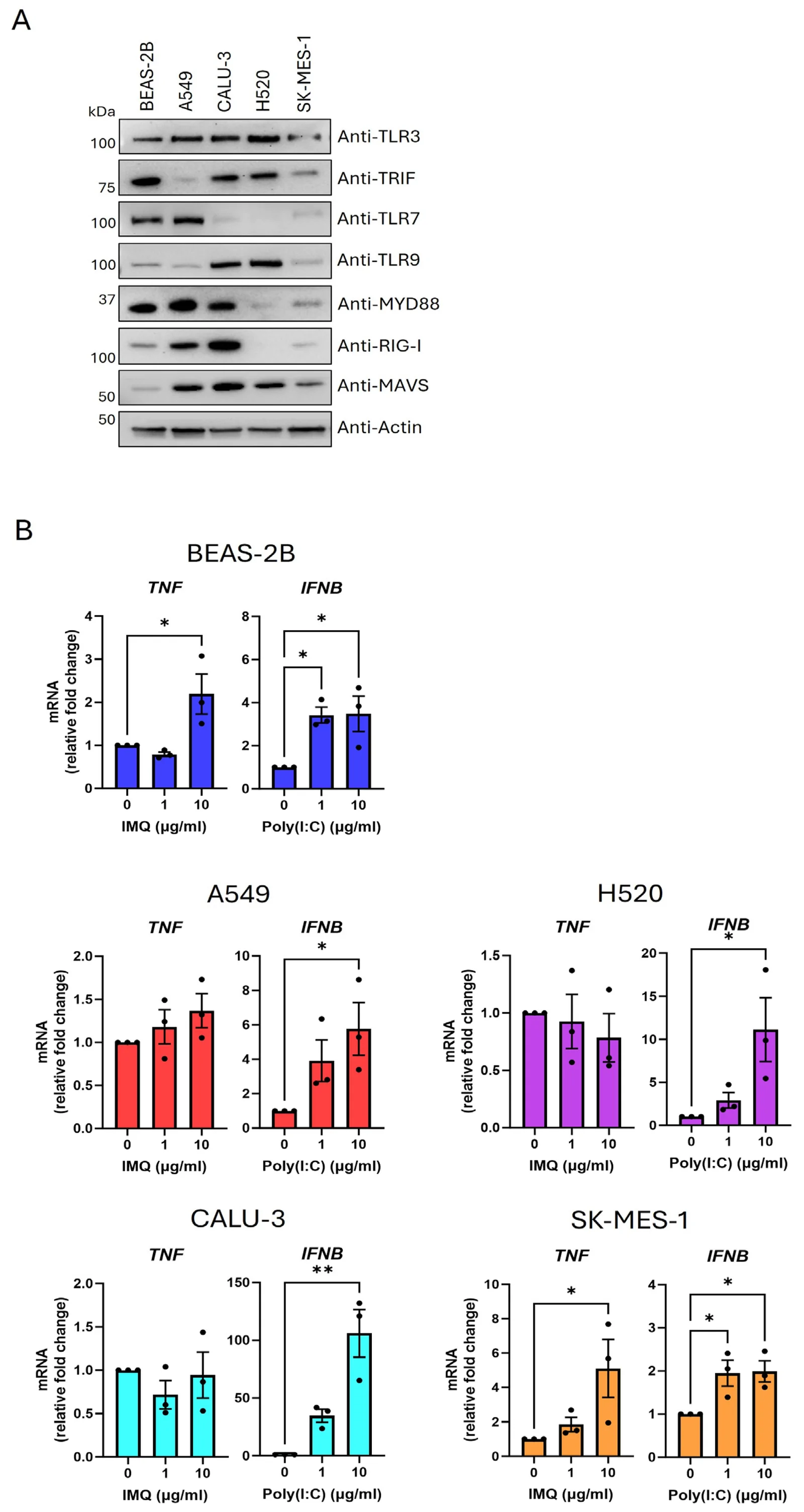
NSCLC cells exhibit distinct basal PRR pathway protein expression and functional activation. (**A**) Western blotting was performed to measure the basal expression of various PRRs and adaptor proteins in malignant A549, CALU-3, H520 and SK-MES-1 and non-malignant BEAS-2B lung epithelial cell lines. Western blot data are representative of a single experiment. (**B**) Lung epithelial cell lines were treated with TLR7 agonist imiquimod (IMQ) or TLR3 ligand poly(I:C) for 6 h and cytokine gene expression was measured by RT-qPCR. Data are presented relative to 18S housekeeping as a fold-change above uninfected controls (2^−ΔΔCt^). Data are expressed as mean ± SEM from 3 independent biological replicates. Statistical analysis was conducted using a one-way ANOVA test followed by Dunnett’s post hoc test for multiple comparisons (* *p* < 0.05, ** *p* < 0.01). The original image of western blot are shown in Supplementary File S1.

The observed variability in cytokine induction and viral abundance suggested some underlying differences exist among the cell lines in terms of their viral sensing capacity. We therefore profiled basal expression of key PRRs and adaptor proteins involved in IAV detection (Figure 5A). TLR3 is an endosomal receptor that senses double-stranded RNA to induce NF-κB signalling and type I IFN production. TLR3 was expressed comparably across cell lines. Its adaptor TRIF was also detected but A549 and SK-MES-1 exhibited low expression. TLR7 is an endosomal receptor that senses ssRNA and activates NF-κB signalling and type I IFN production. TLR7 was expressed in BEAS-2B and A549 cells, faint in CALU-3 and SK-MES-1 cells, and absent in H520 cells. TLR9 is an endosomal receptor that senses unmethylated DNA. TLR9 was expressed in CALU-3 and H520 cells, with low expression in the other epithelial cells. The TLR7/TLR9 adaptor MYD88 was expressed in BEAS-2B, A549 and CALU-3 cells, with low expression in H520 and SK-MES-1 cells. Basal RIG-I expression, which detects RNA present in the cytosol, was evident in A549 and CALU-3 cells, lower in BEAS-2B and SK-MES-1 cells, and undetectable in H520 cells. Expression of the adaptor MAVS was observed in all cell lines with lower detection in BEAS-2B and SK-MES-1 cells. Together, these findings indicate that the lung epithelial cells analysed in this study exist in distinct basal innate immune states, characterised by differential PRR and adaptor expression profiles.

To assess functional competence, cells were stimulated with imiquimod (IMQ), a TLR7 agonist, or poly(I:C), a ligand for TLR3 (Figure 5B). Stimulation of these receptors activates NF-κB- and/or IRF-mediated cytokine production. IMQ induced *TNF* upregulation in BEAS-2B and SK-MES-1 cells, but not in A549 cells despite detectable TLR7 protein, revealing a disconnect between receptor expression and downstream signalling. In contrast, poly(I:C) induced *IFNB* in all cell lines, indicating intact TLR3 signalling capacity.

Together, these data demonstrate substantial heterogeneity in both PRR abundance and downstream signalling among the lung epithelial cells. We also identify evidence of specific functional uncoupling between receptor expression and signalling competence in a subset of cells. This uncoupling suggests that some tumour cells may be unable to consistently translate viral recognition into effective antiviral responses.

### 3.4. IAV Induces Heterogeneous Regulation of Antiviral Sensing Pathways Across NSCLC Cell Lines

**Figure 6:**
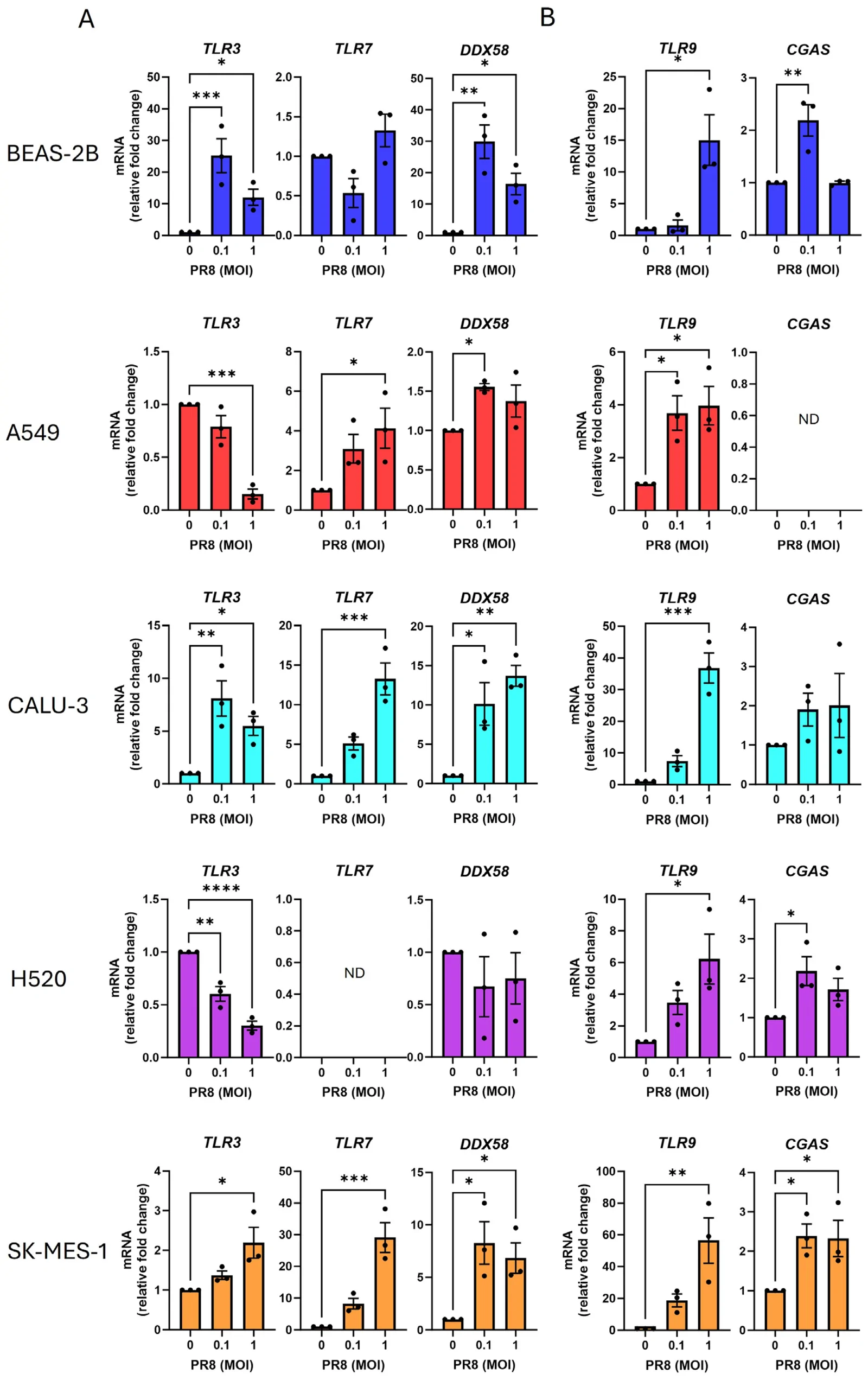
IAV regulates expression of antiviral sensing pathways in NSCLC cells. Malignant A549, CALU-3, H520 and SK-MES-1 and non-malignant BEAS-2B lung epithelial cell lines were infected with PR8 virus at MOI of 0, 0.1 and 1 for 24 h. Gene expression of (**A**) RNA sensors and (**B**) DNA sensors were measured by RT-qPCR. Data are presented relative to 18S housekeeping as a fold-change above uninfected controls (2^−ΔΔCt^). Genes that failed to reach cycle threshold across the sample group and were below the limit of quantification are denoted as “ND” (not detected). Data are expressed as mean ± SEM from 3 independent biological replicates. Statistical analysis was conducted using a one-way ANOVA test followed by Dunnett’s post hoc test for multiple comparisons (* *p* < 0.05, ** *p* < 0.01, *** *p* < 0.001, **** *p* < 0.0001).

Given the observed differences in basal PRR expression and uncoupled signalling competence in some cells, we next investigated how IAV infection dynamically regulates the expression of key viral sensing pathways. We first examined sensors that directly recognise viral RNA species, including endosomal TLR3 and TLR7 and cytosolic RIG-I (encoded by *DDX58*) (Figure 6A). *TLR3* transcripts increased significantly in BEAS-2B, CALU-3 and SK-MES-1 cells following infection, whereas significant downregulation was observed in A549 and H520 cells. *TLR7* expression increased in A549, CALU-3 and SK-MES-1 cells. *TLR7* expression was undetectable in H520 cells under both basal and infected conditions. Infection induced significant upregulation of *DDX58* in all cell lines except H520 cells. These findings indicate that RNA-sensing pathways are inconsistently regulated across the lung epithelial cell lines following infection.

We next assessed DNA-sensing pathways, including endosomal TLR9 and the cytosolic sensor cGAS (Figure 6B). In contrast to the heterogeneous regulation of RNA-sensing PRRs, *TLR9* was significantly upregulated across all cell lines, irrespective of basal expression. Similarly, *CGAS* expression increased in BEAS-2B, H520 and SK-MES-1 cells, but remained undetectable in A549 cells. These findings suggest that stress-associated DNA-sensing receptors were transcriptionally upregulated during infection, even in the context of the variable RNA-sensing responses.

Together, these data demonstrate that IAV infection induces specific alteration of antiviral sensing pathways in the lung epithelial cell lines we tested, characterised by inconsistent regulation of canonical RNA sensors alongside conserved induction of selected stress-associated DNA-sensing genes. Among the NSCLC cells, this pattern of response suggests that antiviral sensing is not uniformly amplified and may account for some of the sustained accumulation of viral content as observed earlier.

### 3.5. IAV-Associated PRR Regulation in Lung Epithelial Cells Involves IFNAR1-Dependent and -Independent Mechanisms

**Figure 7:**
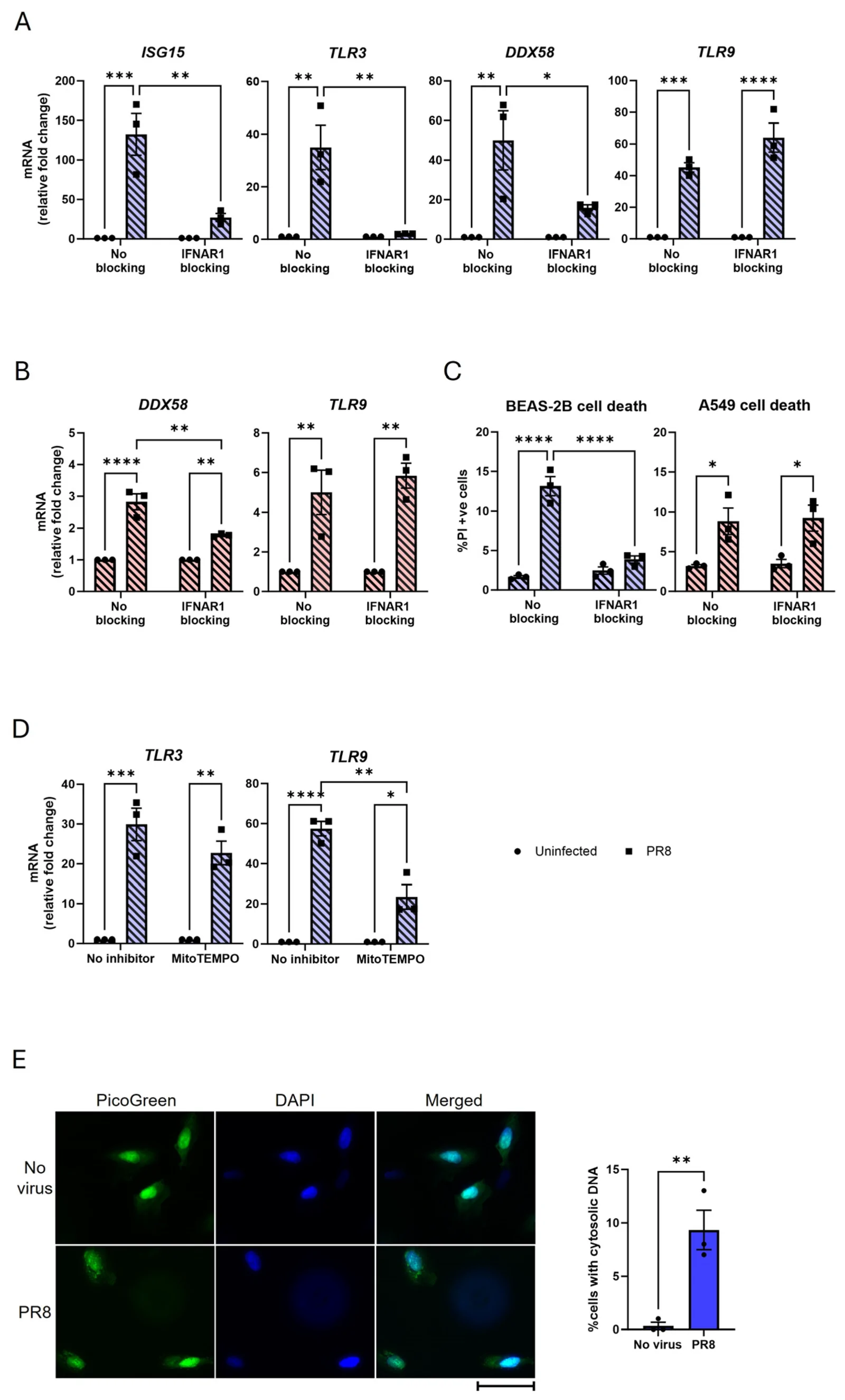
IFN-dependent or ROS-associated signalling pathways in lung epithelial cells are involved in antiviral sensing. Lung epithelial cells were incubated with 0.5 µg/ml of IFNAR1 antibody for 1 h prior to infection with PR8 virus (MOI of 1). Gene expression analysis was measured by RT-qPCR after 24 h in (**A**) non-malignant BEAS-2B and (**B**) malignant A549 cells. (**C**) Cell death was also measured by PI uptake assays. (**D**) Non-malignant BEAS-2B cells were treated with 50 µM mitoTEMPO for 1 h prior to infection with PR8 virus (MOI of 1). Gene expression analysis was then measured by RT-qPCR after 24 h. Gene expression data are presented relative to 18S housekeeping as a fold-change above uninfected controls (2^−ΔΔCt^). (**E**) PicoGreen (dsDNA) and DAPI (nuclear) staining of BEAS-2B cells following 24 h infection with PR8 virus (MOI of 1) was visualised by microscopy. Scale bar represents 50 µm. The percentage of cells with punctate cytosolic PicoGreen staining was determined upon counting 100 cells in random fields of view. Data are expressed as mean ± SEM from 3 independent biological replicates. Statistical analysis was conducted using (**A**–**D**) a two-way ANOVA test followed by Tukey’s post hoc test for multiple comparisons or (**E**) an unpaired *t*-test (* *p* < 0.05, ** *p* < 0.01, *** *p* < 0.001, **** *p* < 0.0001).

To define the regulatory mechanisms underlying differential PRR induction following infection, IFNAR1 signalling was blocked prior to virus exposure in BEAS-2B cells (Figure 7A). Inhibition of IFN signalling reduced *ISG15* induction, confirming effective pathway blockade. Under these conditions, infection-mediated upregulation of *TLR3* and *DDX58* was abolished, which is consistent with IFN-dependent regulation. In contrast, *TLR9* induction was unaffected, indicating an IFNAR1-independent mechanism. Similar results were observed in A549 cells, where IFNAR1 blockade attenuated virus-induced *DDX58* upregulation but had no effect on *TLR9* expression (Figure 7B). IFNAR1 inhibition also reduced infection-induced cell death in BEAS-2B but not A549 cells (Figure 7C), suggesting that type I IFN signalling contributes to apoptotic sensitivity in non-malignant BEAS-2B cells.

Given the IFN-independent induction of *TLR9*, we next investigated whether mitochondrial stress contributes to its regulation. TLR9 recognises unmethylated DNA motifs and mitochondrial DNA (mtDNA) represents a potential endogenous ligand under conditions of cellular stress. To test this, mitochondrial reactive oxygen species (ROS) were scavenged using mitoTEMPO. This treatment partially attenuated *TLR9* upregulation without affecting *TLR3* expression (Figure 7D), implicating mitochondrial ROS in the selective regulation of *TLR9* expression. We also detected increased cytosolic DNA abundance following infection (Figure 7E). While we do not identify whether the DNA species originates from the mitochondria or nucleus, these observations suggest that the abundance of DNA throughout the cell and accompanying increase in DNA-sensing receptor expression provoked by infection could potentially enhance activation of a specific DNA-sensing pathway.

Together, these findings demonstrate that IAV-associated regulation of selected PRRs can occur through IFNAR1-dependent and IFNAR1-independent mechanisms. Along with enhanced cytosolic DNA, these findings identify a potential virus-induced stress-associated mechanism for PRR regulation in lung epithelial cells.

### 3.6. IAV-Associated Type I IFN Signalling Contributes to Immune Checkpoint Upregulation in Lung Epithelial Cells

**Figure 8:**
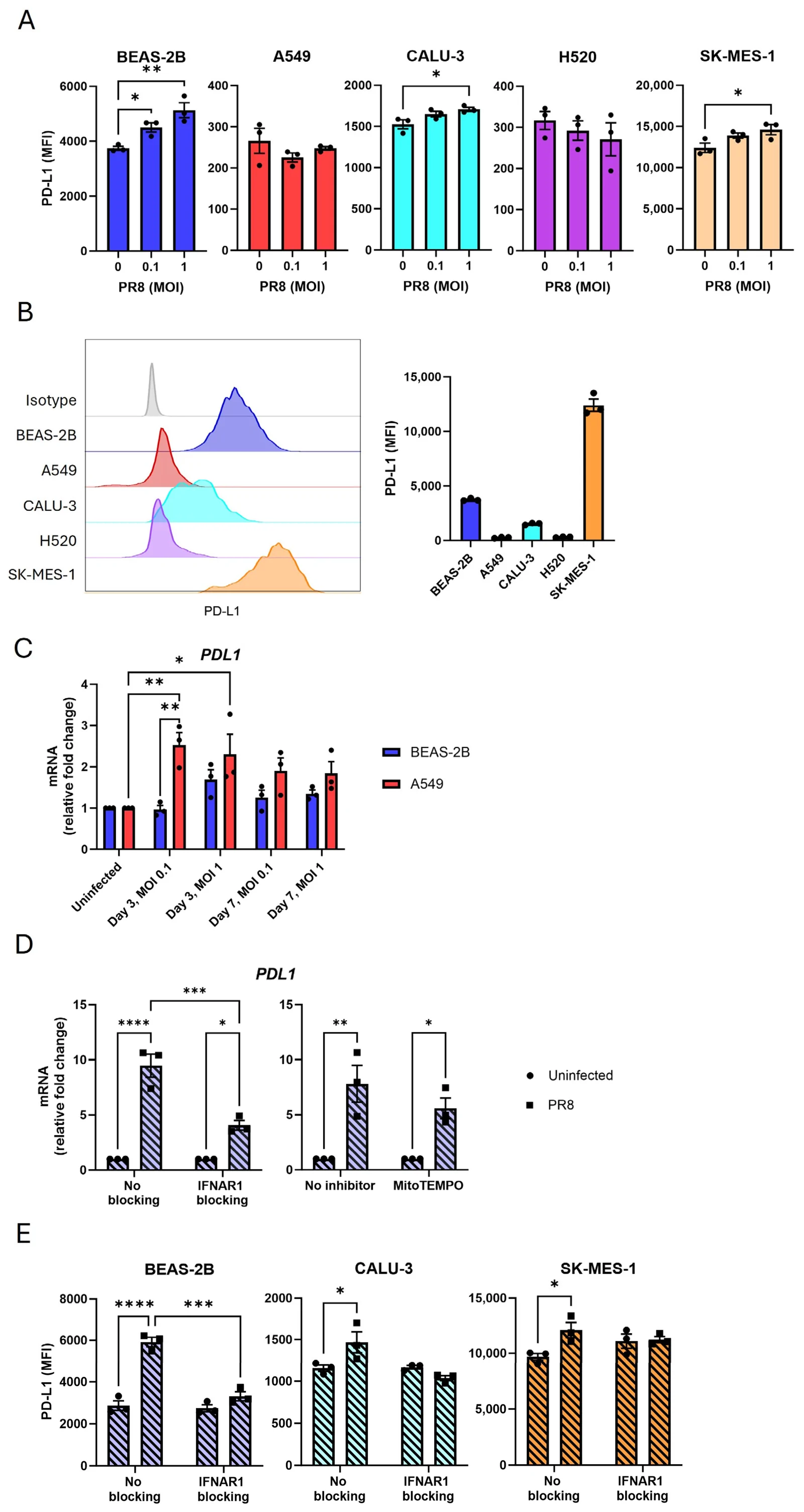
IAV induces immune checkpoint upregulation in lung epithelial cells via IFN pathways. Malignant A549, CALU-3, H520 and SK-MES-1 and non-malignant BEAS-2B lung epithelial cell lines were infected with PR8 virus at MOI of 0, 0.1 and 1 for 24 h. (**A**) Cell surface protein expression of PD-L1 was measured by flow cytometry and is presented as mean fluorescent intensity (MFI). (**B**) Comparison of basal PD-L1 expression in uninfected lung epithelial cells is shown. (**C**) Gene expression analysis of infected BEAS-2B and A549 cells was also performed at 3 and 7 days post-infection. Cells were incubated with 0.5 µg/ml of IFNAR1 antibody or 50 µM mitoTEMPO for 1 h prior to infection with PR8 virus (MOI of 1) for 24 h. (**D**) *PDL1* transcripts in BEAS-2B cells, or (**E**) PD-L1 cell surface expression in BEAS-2B, CALU-3 or SK-MES-1 cells were measured. Gene expression data are presented relative to 18S housekeeping as a fold-change above uninfected controls (2^−ΔΔCt^). Data are expressed as mean ± SEM from 3 independent biological replicates. Statistical analysis was conducted using (**A**) a one-way ANOVA test followed by Dunnett’s post hoc test, or a two-way ANOVA test followed by (**C**) Sidak’s post hoc test for multiple comparisons to compare between cell lines (**D**,**E**) or Tukey’s post hoc for multiple comparisons (* *p* < 0.05, ** *p* < 0.01, *** *p* < 0.001, **** *p* < 0.0001).

Given that the lung epithelial cells in this study retained IFN signalling accompanied by viral abundance, we next examined whether this preserved antiviral response influences expression of immune checkpoint ligands. Transcriptional analysis revealed that *PDL1* (*CD274*) was significantly upregulated following infection in BEAS-2B, A549, CALU-3 and SK-MES-1 cells (Supplementary Figure S4). In contrast, H520 cells showed no induction of *PDL1*. Expression of *PDL2* (*PDCD1LG2*) followed a similar pattern, with induction observed in all cell lines except H520, in which *PDL2* was undetectable under both basal and infected conditions. Analysis of cell surface PD-L1 expression generally mirrored the transcriptional findings, with BEAS-2B, CALU-3, and SK-MES-1 cells exhibiting significant increases in surface PD-L1 following infection (Figure 8A). In comparison, A549 and H520 cells expressed little basal PD-L1 surface expression (Figure 8B) and did not exhibit significant infection-induced upregulation at this timepoint, highlighting differences in basal PD-L1 surface expression among lung epithelial cell lines. Notably, despite the absence of an early increase in surface PD-L1, *PDL1* transcript levels in A549 cells were significantly elevated three days after IAV infection (Figure 8C). In contrast, infection-induced *PDL1* upregulation in BEAS-2B cells was not maintained at these later timepoints. These findings indicate that IAV infection promotes immune checkpoint ligand expression in most lung epithelial cell lines and this response may persist alongside prolonged infection in some cells.

To investigate the mechanism underlying PD-L1 induction, we assessed the contribution of IFN signalling and mitochondrial ROS. Blockade of IFNAR1 prior to infection significantly reduced *PDL1* upregulation in BEAS-2B cells (Figure 8D), indicating that type I IFN signalling is a primary driver of PD-L1 expression following IAV infection. In contrast, scavenging mitochondrial ROS with mitoTEMPO had no significant effect on *PDL1* induction. Consistent with the transcriptional data, IFNAR1 blockade also reduced infection-induced surface PD-L1 expression in BEAS-2B, CALU-3 and SK-MES-1 cells (Figure 8E).

Together, these findings demonstrate that type I IFN signalling contributes to acute IAV-associated PD-L1 upregulation in the non-malignant and NSCLC cell lines used in this study.

## 4. Discussion

This study investigated tumour cell-intrinsic antiviral responses to IAV infection across a panel of lung epithelial cell lines. Our findings demonstrate that NSCLC cells retain the capacity to mount IFN signalling, with these responses associated with viral protein accumulation and induction of downstream pathways linked to immune modulation and cell survival. Type I IFN signalling promoted upregulation of PD-L1, implicating antiviral responses in the tumour immune checkpoint phenotype, and also contributed to infection-induced apoptotic signalling in non-malignant epithelial cells. In contrast, NSCLC cells exhibited relative resistance to acute IAV-induced cell death. Together, these findings highlight cell line-associated heterogeneity in NSCLC responses to IAV, encompassing antiviral signalling, cell death susceptibility and immune-modulatory responses. This variability supports the concept that malignant transformation may reshape tumour cell-intrinsic antiviral responses, differentially influencing viral restriction, cell survival and inflammatory signalling, with potential consequences for the tumour immune landscape following viral infection. Such heterogeneity may reflect, in part, differences in how individual tumour cell populations respond to infection, thereby contributing to the context-dependent effects of respiratory viral infection on tumour biology.

Mounting an IFN response is critical to antiviral cytokine production that restricts viral replication, and all of the cell lines examined here induced either or both type I and III IFNs following infection. Our findings are consistent with previous reports showing elevated basal IFN signalling in non-malignant BEAS-2B cells compared with transformed lung epithelial lines [21] and with high levels persisting 7 days after infection. In contrast, A549 adenocarcinoma cells, which reportedly support higher levels of viral replication, lacked detectable *IFNL* induction despite robust acute *IFNB* responses, suggesting selective impairment of antiviral IFN programmes that may favour infectivity and viral persistence in this particular tumour cell. Indeed, unlike non-malignant BEAS-2B cells, these A549 cells could not sustain *IFNB* upregulation at later timepoints, and this was accompanied by higher viral RNA abundance. Such variability may reflect tumour-associated alterations in IFN signalling, as some NSCLC tumours harbour mutations affecting IFN gene expression and downstream ISG responses [22]. Importantly, viral transcripts and nucleoprotein were detected in all cell lines, with sustained accumulation of viral protein observed in NSCLC cells despite intact acute IFN signalling. However, the detection of viral RNA and protein alone does not establish whether these signals reflect ongoing productive infection, delayed clearance of viral material, or another form of prolonged infection-associated state. Longitudinal studies incorporating measurements of infectious viral output will therefore be important to determine the extent to which sustained viral signals in NSCLC models reflect continued productive infection and to clarify the relationship between viral persistence and tumour cell-intrinsic antiviral responses. These findings suggest that, while antiviral signalling pathways remain broadly active in NSCLC, they may be insufficient to effectively restrict infection, at least in some cell lines, with tumour-specific differences in IFN responses potentially influencing viral abundance, PRR engagement and downstream immune-modulatory signalling within infected tumour cells.

A key consequence of the IFN signalling in most of the cells following IAV infection was the upregulation of PD ligands. This extends previous observations in A549 cells [23] and demonstrates that infection-driven immune checkpoint induction is a feature across multiple NSCLC cell lines. PD-L1 induction appeared to be most evident in cells mounting high *IFN* fold changes and was largely dependent on IFNAR1 signalling, which is consistent with established IFN–STAT regulation of PD-L1 and PD-L2 expression in NSCLC [24,25]. In contrast, cells with weaker *IFN* fold changes that were not as high, such as H520, failed to upregulate PD-L1, suggesting that checkpoint upregulation is closely linked to the magnitude of retained antiviral signalling. PD-L1 surface expression remained unchanged in infected A549 cells, which, unlike the other cell lines, also lacked an *IFNL* response and this potentially implicates type III IFNs in PD-L induction. Because IFNAR1 blockade experiments specifically interrogate type I IFN signalling, the potential contributions from type III IFNs remain to be determined in this context. The overall findings align with in vivo observations that IAV infection enhances PD-L1 expression in lung cells through a mechanism requiring TLR7-dependent cytokine signalling [26], highlighting an additional layer of regulation linking viral sensing to immune checkpoint upregulation. Given that IAV infection results in differential TLR expression in NSCLC cells, our data suggest that IFN-driven and TLR-mediated pathways converge to upregulate PD-L1 expression within infected tumour cells. Importantly, the persistence of apoptosis-resistant infected tumour cells may prolong these signalling responses over time, allowing prolonged immune checkpoint ligand expression during sustained infection-associated signalling to potentially further impair anti-tumour immune responses. As such, we found that *PDL1* transcripts were upregulated days after infection in malignant A549 cells compared to non-malignant BEAS-2B cells. However, *IFNB* levels were lower in comparison to BEAS-2B cells at these same timepoints, suggesting that prolonged *PDL1* increase may not necessarily be dependent on sustained *IFNB* levels, at least in A549 cells, and may involve other mechanisms to maintain persistence. These findings support a model in which NSCLC cells retain selective antiviral signalling outputs that potentially contribute to an immune-modulatory phenotype despite accumulating viral material. Supported by prior functional evidence that IAV-induced upregulation of PD-L1 led to suppression of cytotoxic T cell responses in vivo to enhance tumour progression [13,14], this virus–tumour signalling nexus may represent a conserved mechanism linking respiratory viral infection to tumour immune escape.

We also propose that the observed resistance to acute IAV-induced cell death in NSCLC cells may have important functional consequences, as elimination of infected epithelial cells is a key mechanism for restricting viral replication and terminating inflammatory signalling [27]. NSCLC frequently exhibit elevated expression of anti-apoptotic BCL-2 family proteins, including MCL-1 and BCL-xL, which promote tumour cell survival and resistance to a range of cellular stresses [28,29]. Such a pro-survival phenotype may limit IAV-induced apoptosis, thereby prolonging the viability of infected tumour cells and potentially extending the window for viral replication and persistence. This prolonged survival may also permit continued cytokine production [30] to create a persistent inflammatory signalling state within the tumour microenvironment. Indeed, differential induction of *IL6* and *TNF* across cell lines suggests that infection may variably promote pro-tumourigenic inflammation depending on the tumour. IL-6 and TNF promote tumour-associated inflammation, survival signalling and immune modulation in lung cancer [31], and the infection of tumour cells may provide a persistent source of pro-tumourigenic cytokines. Thus, our findings provide further insight into the potential consequences of resistance to infection-induced cytotoxicity, as it may prolong infection-associated signalling and contribute to a sustained inflammatory state that could support viral persistence and tumour-associated inflammatory remodelling. However, these potential tumour-promoting effects should be considered alongside evidence that IAV infection can also exert direct oncolytic and anti-tumour effects in lung cancer models. For instance, Masemann, Köther, Kuhlencord, Varga, Roth, Lichty, Rapp, Wixler and Ludwig [20] demonstrated substantial IAV-mediated oncolysis in a spontaneous mouse model of NSCLC, accompanied by macrophage reprogramming towards an inflammatory, anti-tumour phenotype. Similarly, IAV infection prior to tumour establishment has been reported to enhance immune surveillance and reduce tumour burden [18,19]. These contrasting outcomes highlight the context dependence of the virus–tumour interaction, whereby IAV-induced inflammation and tumour cell death may promote tumour restriction in some settings, while sustained signalling in apoptosis-resistant tumour cells may instead favour tumour-promoting inflammation during established disease. The balance between these effects is likely shaped by the timing of infection, tumour stage, tumour cell-intrinsic antiviral and survival responses, and the surrounding immune microenvironment.

The heterogeneity in cytokine responses in lung cancer cells was associated with differences in PRR expression and signalling competence. Notably, receptor abundance did not always predict responsiveness, as exemplified by TLR7 expression in A549 cells in the absence of functional downstream signalling. This observation is consistent with previous reports demonstrating heterogeneous TLR7 responsiveness in A549 cells. While some studies have shown that TLR7 agonists can activate NF-κB signalling to promote cell survival pathways, others have reported minimal NF-κB activation and cytokine production following stimulation with IMQ, resiquimod (R848) or related agonists [6,32,33]. Furthermore, IMQ has been shown to exert antiviral effects in A549 cells through mechanisms independent of canonical TLR7 signalling, including activation of protein kinase A pathways [33]. Together, these findings suggest that TLR7 signalling in A549 cells may be selectively uncoupled from classical inflammatory cytokine responses or exhibit ligand-specific signalling outputs. This reinforces the concept that downstream signalling capacity, rather than receptor abundance alone, determines how tumour cells interpret and respond to viral infection. Such uncoupling may arise from oncogenic rewiring of innate immune pathways in NSCLC, where dysregulation of NF-κB, STAT or IRF signalling networks, as well as metabolic or epigenetic alterations associated with malignant transformation, can alter the functional output of PRR activation. Importantly, PRR signalling in tumour cells can exert context-dependent effects on cancer progression. Elevated TLR7 expression has been associated with tumour-intrinsic pro-tumourigenic signalling in NSCLC [6,7], whereas higher TLR3 expression in lung adenocarcinoma correlates with improved patient survival [34,35]. TLR9 is also frequently expressed in lung tumours [36], although its expression does not consistently correlate with favourable survival outcomes [37]. Although our findings identify substantial differences in antiviral receptor expression and downstream inflammatory responses across NSCLC cell lines, they do not provide a comprehensive mapping of the signalling pathways responsible for these differences. Functional agonist experiments and targeted perturbation of IFNAR1 signalling and mitochondrial ROS provide evidence for the involvement of selected pathways, but do not establish the complete molecular mechanisms linking individual receptors to downstream transcriptional responses. Future studies incorporating quantitative analyses of transcription factor activation and targeted genetic approaches will be important for defining the signalling networks underlying these heterogeneous responses. Further in-depth analysis is also needed to establish whether malignant transformation itself rewires antiviral immunity, although our findings suggest that NSCLC cells may exist in distinct basal innate immune states that shape whether antiviral sensing pathways promote effective viral restriction or sustained inflammatory signalling.

RNA-sensing receptors (TLR3, TLR7 and DDX58) are central to antiviral recognition and the restriction of virus propagation, and we showed that IAV infection induced variable regulation of nucleic acid-sensing pathways in lung cancer cells. In contrast, DNA-sensing receptor expression was more uniformly engaged, with consistent induction of TLR9 and broader upregulation of cGAS, suggesting preferential modulation of conserved stress-associated sensing mechanisms during infection. Importantly, sensing of self-DNA, including mtDNA released during cellular stress, is a recognised driver of inflammatory responses and contributes to the pathogenesis of inflammatory lung disease [38]. Mechanistically, our findings suggest that RNA- and DNA-sensing pathways exhibit differential regulation during IAV infection in lung epithelial cells, rather than representing strictly separated IFN- versus ROS-dependent pathways. RNA sensor induction was strongly influenced by IFNAR1 signalling, whereas TLR9 upregulation occurred independently of IFNAR1 and was partially mediated by mitochondrial ROS. Consistent with this, IAV infection increased cytosolic DNA accumulation, supporting the possibility that infection-associated cellular stress contributes to regulation of DNA-sensing receptor expression. Given that mitochondrial dysfunction and oxidative stress can promote the release of endogenous DNA species, including mtDNA, these findings suggest that mitochondrial stress may provide an additional source of innate immune stimulation during persistent infection. Furthermore, DNA can also be sensed by the cytosolic cGAS-STING platform following IAV infection to activate NF-κB responses [39]. These findings suggest that antiviral sensing in NSCLC cells becomes differentially regulated during infection, with variable engagement of pathways associated with viral sensing and stress-associated inflammatory sensing pathways. However, the precise identity and origin of the cytosolic DNA species that could signal through TLR9, cGAS-STING or alternative pathways requires further investigation to define the contribution of these findings.

Given the capacity for IAV infection to disrupt mitochondrial homeostasis and increase ROS production [40], we therefore propose that mitochondrial ROS may contribute to TLR9 upregulation through redox-sensitive signalling pathways, potentially involving NF-κB or ERK, which have previously been implicated in the regulation of TLR9 expression [41]. In parallel with IAV-induced mtDNA release providing endogenous activation of DNA-sensing pathways, including TLR9 and cGAS [42,43,44], we speculate that these processes could establish a feed-forward response in which mitochondrial stress promotes TLR9 expression, while DNA-sensing and downstream inflammatory signalling further reinforce this response. Indeed, TLR9 deficiency reduced acute inflammation in a mouse model of IAV infection but resulted in higher viral burden and worse disease outcomes [45], highlighting a complex balance between antiviral defence and immunopathology. Moreover, increased circulating mtDNA and enhanced TLR9 activation have been observed in patients with IAV infection [45] or separately in lung cancer [46], further supporting the clinical relevance of this pathway. Given that mitochondrial dysfunction and oxidative stress are common features in tumour cells, a context of persistent viral infection within NSCLC cells may provide a favourable environment for sustained activation of ROS-dependent DNA-sensing pathways. In this setting, TLR9 activation may represent a conserved mechanism linking unresolved viral infection to chronic inflammatory signalling in NSCLC. Importantly, these stress-associated DNA-sensing pathways can be engaged alongside canonical IFN-mediated antiviral sensing, suggesting that tumour cells integrate antiviral and inflammatory signals through multiple interconnected pathways during persistent infection. Together, these findings support a model in which IAV infection establishes an innate immune state within tumour cells, characterised by upregulation of IFN-dependent immune-modulatory pathways and stress-associated DNA sensing, which may contribute to sustained viral infection and inflammatory signalling.

A limitation of this study was the use of in vitro epithelial models, which do not fully recapitulate the complexity of the tumour microenvironment. In addition, the non-malignant comparator used in this study, BEAS-2B cells, are SV40-immortalised bronchial epithelial cells and therefore may not fully reflect the antiviral signalling and stress responses of primary airway epithelium. Nevertheless, this system models how infection of established NSCLC cells may directly influence tumour cell-intrinsic antiviral and inflammatory signalling. Previous studies have reported broadly similar responses to IAV infection in BEAS-2B and primary normal human bronchial epithelial (NHBE) cells when compared with A549 cells, despite elevated basal expression of certain IFN-stimulated genes in BEAS-2B cells [21]. Furthermore, A549 cells have been shown to exhibit greater resistance to IAV-induced cell death than NHBE cells [27], suggesting that the differential responses observed in the non-malignant and NSCLC cells in the present study may extend to primary epithelial models. Future studies incorporating primary airway epithelial cells and more complex in vivo systems will be important to further establish the physiological and translational relevance of these findings and how tumour heterogeneity contributes to the variable outcomes of respiratory viral infection.

Importantly, previous in vivo studies demonstrate that intranasally delivered IAV can directly infect tumour cells in the lungs and modulate tumour progression by effects on immune cells, including macrophages and T cells [13]. Tumour-associated macrophages are key regulators of lung tumour progression and tissue-resident macrophages can promote early tumour development in the lungs by enhancing epithelial–mesenchymal transition and invasiveness [47]. Viral infection may further modulate these interactions, as prior IAV infection can “train” alveolar macrophages to enhance phagocytic and anti-tumour functions [19], whereas infection during established NSCLC may promote tumour growth through PD-L1-mediated suppression of cytotoxic T cell responses [13]. Our findings suggest that tumour cells themselves may contribute directly to these processes via cell-intrinsic responses to viral infection. In this context, sustained checkpoint expression and cytokine production from infected, apoptosis-resistant tumour cells may reinforce local immune suppression and inflammatory remodelling during unresolved infection. Additional studies employing primary airway epithelial–immune cultures, patient-derived NSCLC models, and in vivo tumour systems will therefore be essential to determine how tumour-intrinsic antiviral sensing integrates with immune and stromal components of the tumour microenvironment to influence potential viral persistence, inflammatory pathology and tumour progression.

## 5. Conclusions

Our findings demonstrate substantial heterogeneity in tumour cell-intrinsic responses to IAV across lung epithelial cells, linking respiratory viral infection with differential regulation of antiviral signalling, cell death and immune-modulatory pathways in NSCLC cells. The retention of antiviral signalling alongside reduced susceptibility to infection-induced cell death and increased PD-L1 expression suggests that tumour cell-intrinsic responses may influence the balance between viral restriction, inflammatory signalling and immune regulation following infection. From a translational perspective, these findings provide a foundation for investigating how differences in tumour antiviral and survival responses contribute to the context-dependent effects of respiratory viral infections on cancer progression. Further mechanistic studies using physiologically relevant models and in vivo tumour systems are needed to establish the functional consequences of these responses and determine their relevance to tumour–immune interactions and clinical outcomes.

## Data Availability

The original contributions presented in the study are included in the article material; further inquiries can be directed to the corresponding authors.

## Supplementary Materials

The following supporting information can be downloaded at https://www.mdpi.com/article/10.3390/cancers18213502/s1: Figure S1. Representative flow cytometry gating strategies; Figure S2. Stability of 18S rRNA expression across cell lines and PR8 infection conditions; Figure S3. Uncropped blots relevant to (A) Figure 1C, (B) Figure 3B and (C) Figure 5A in the main text; Figure S4. PD-L gene expression analysis in lung epithelial cells following IAV infection. Supplementary File S1: Original Image of Western Blot.
