# Supplementary material for: Tumour Cell-Associated Heterogeneity in Antiviral Immunity Shapes Influenza Responses and Immune-Modulatory Signalling in Lung Cancer
Source: Cancers (Basel). 2026 Oct 30;18(21):3502. doi: 10.3390/cancers18213502 (PMC13762215; doi:10.3390/cancers18213502)
Supplement: Supplementary file 1 [file cancers-18-03502-s001.zip › cancers-4660199-supplementary/cancers-4660199-Supplementary File S1.pdf]

A549 + PR8 (hours) BEAS-2B + PR8 (hours)

0 2 8 16 24 48 0 2 8 16 24 48

kDa

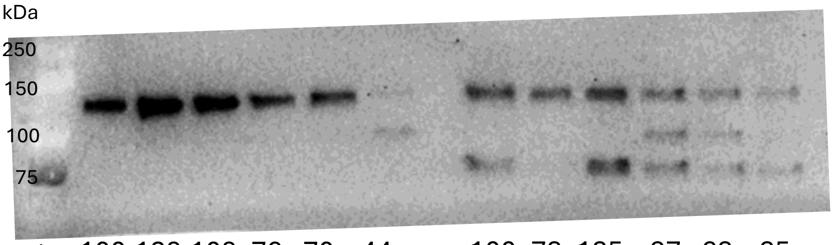

Anti-PARP

Density ratio: 100:128:108: 76: 70: 44 100: 73: 125: 97: 63: 35

kDa

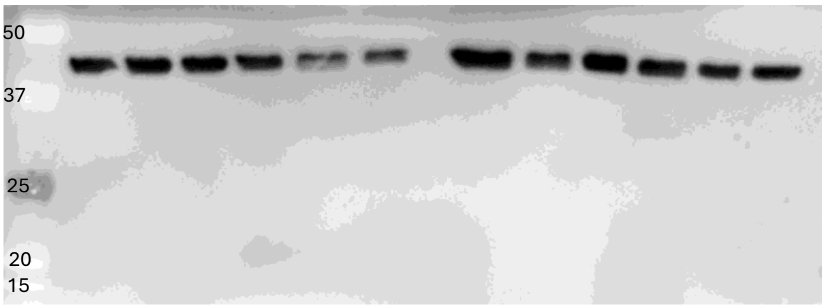

Anti-Actin

Density ratio: 100:123: 139: 147: 96: 86 100: 64: 81: 75: 57: 56

H520 + PR8 (hours)

0 2 8 16 24 48

kDa

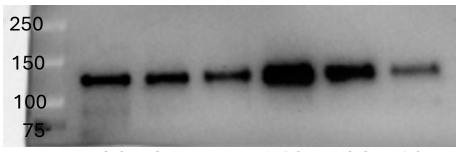

Anti-PARP

Density ratio: 100: 81: 77: 140: 102: 46

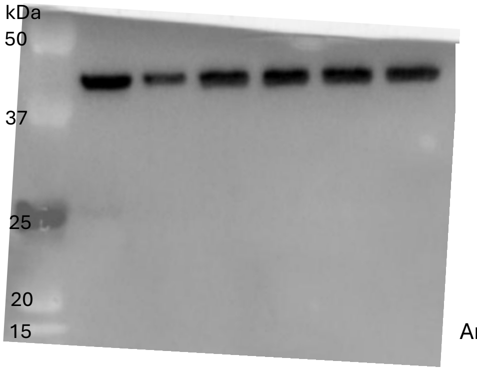

Anti-Actin

Density ratio: 100: 76: 96: 99: 99: 88

Uncropped blots (Figure 1C)

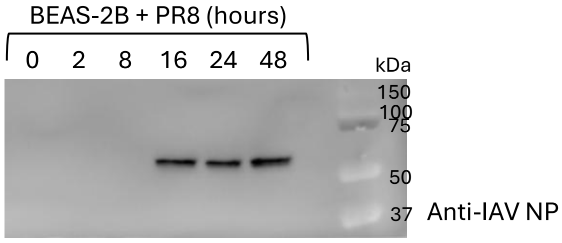

Density ratio: 11:14:27:99:90:100

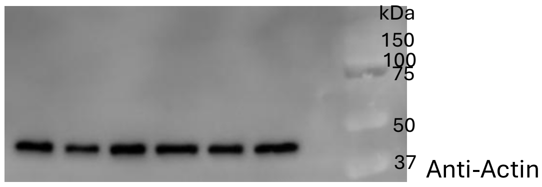

Density ratio: 100:75:105:102:85:103

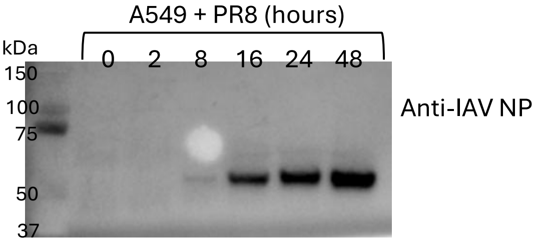

Density ratio: 0.6: 3: 6: 56: 78:100

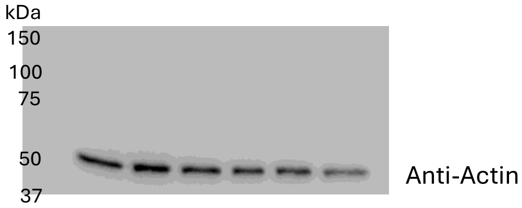

Density ratio: 100:111: 92: 71: 73: 56

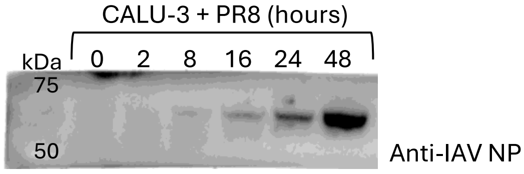

Density ratio: 5: 7: 16: 30: 65:100

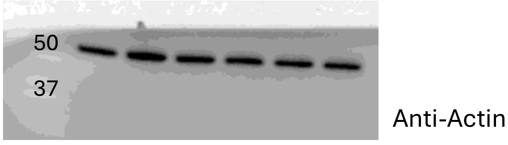

Density ratio:100:147:137:132:126:121

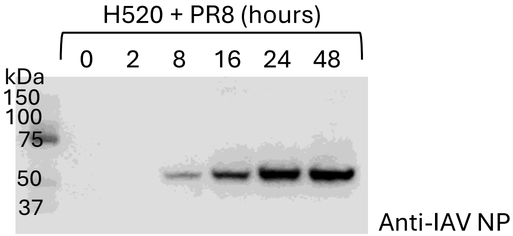

Density ratio: 3.5: 0.9: 21:60: 97: 100

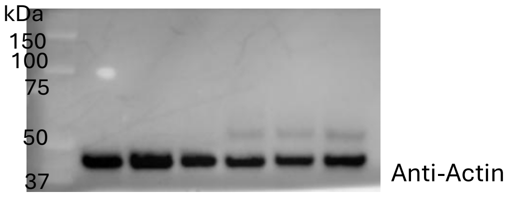

Density ratio: 100:112: 78: 74: 68: 82

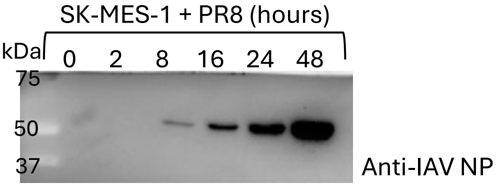

Density ratio: 0.3: 0.3: 1: 16: 41:100

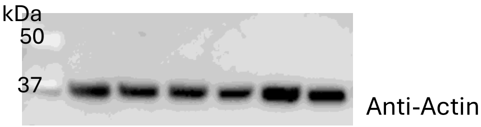

Density ratio: 100:100:98:86:120:104

Uncropped blots (Figure 3B)

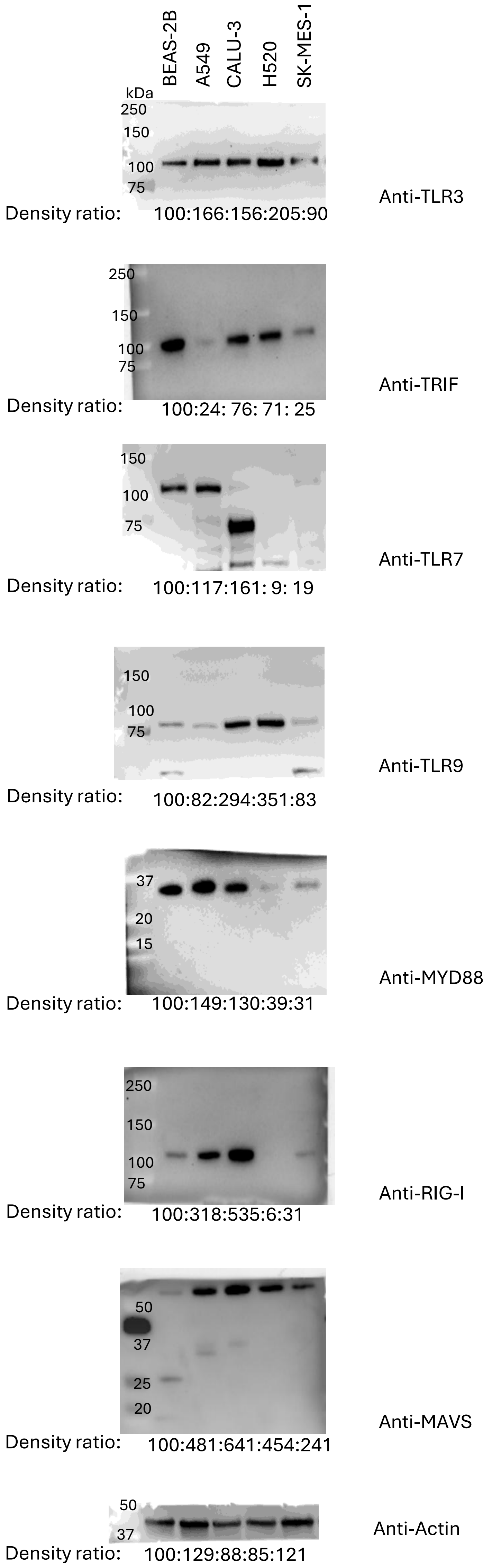

Uncropped blots (Figure 5A)
