# Supplementary material for: Tumour Cell-Associated Heterogeneity in Antiviral Immunity Shapes Influenza Responses and Immune-Modulatory Signalling in Lung Cancer
Source: Cancers (Basel). 2026 Oct 30;18(21):3502. doi: 10.3390/cancers18213502 (PMC13762215; doi:10.3390/cancers18213502)
Supplement: Supplementary file 1 [file cancers-18-03502-s001.zip › cancers-4660199-supplementary/cancers-4660199-supplementary.pdf]

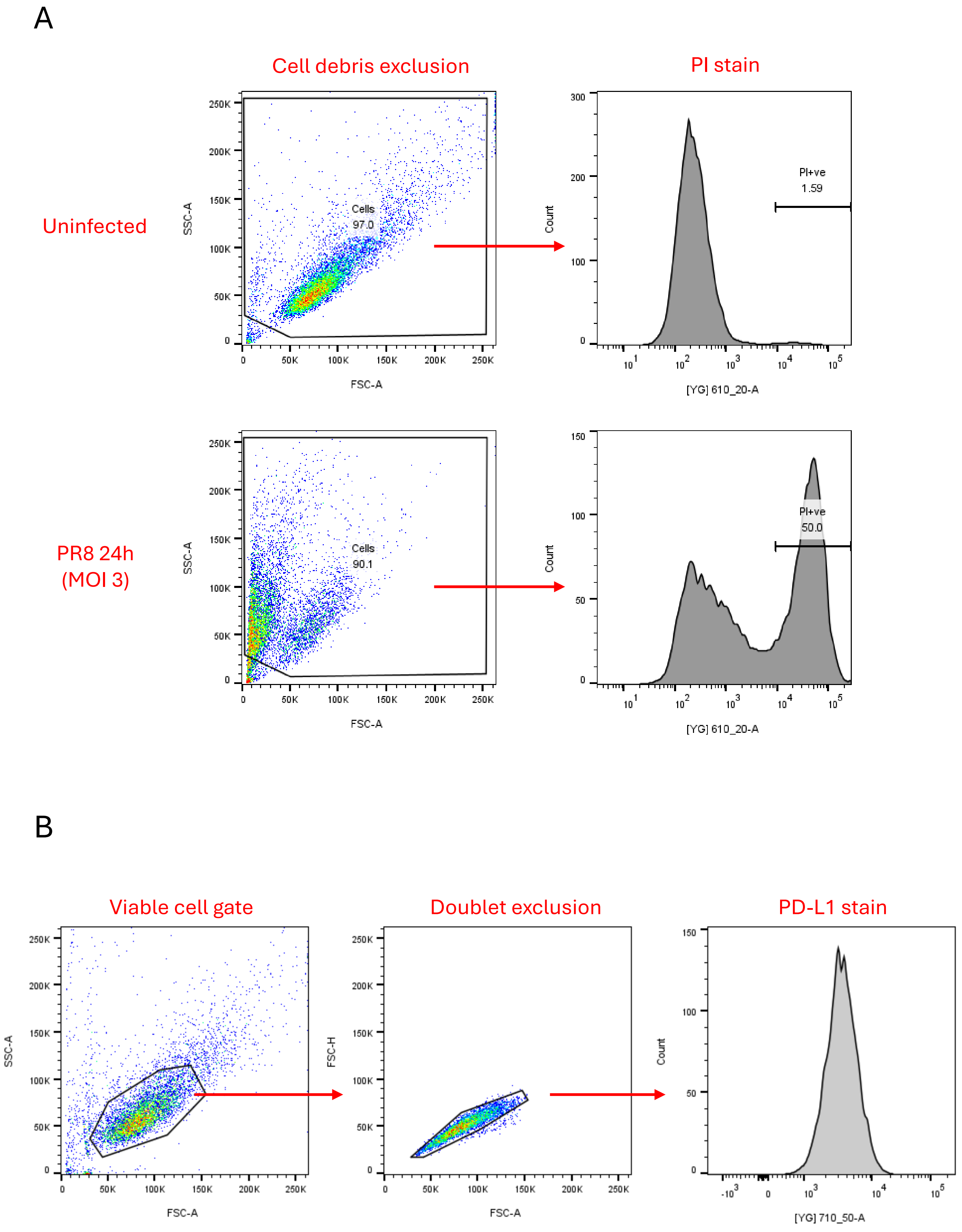

Figure S1. Representative flow cytometry gating strategies. (**A**) For propidium iodide (PI) analysis of cell death, uninfected and infected cells were gated on forward and side scatter to exclude cell debris then the percentage of cells staining positive for PI determined. (**B**) For PD-L1 surface staining, a viable cell gate was created to exclude cell debris and include cells of uniform morphology based on forward and side scatter, followed by exclusion of doublets and the mean fluorescent intensity of PD-L1 staining determined.

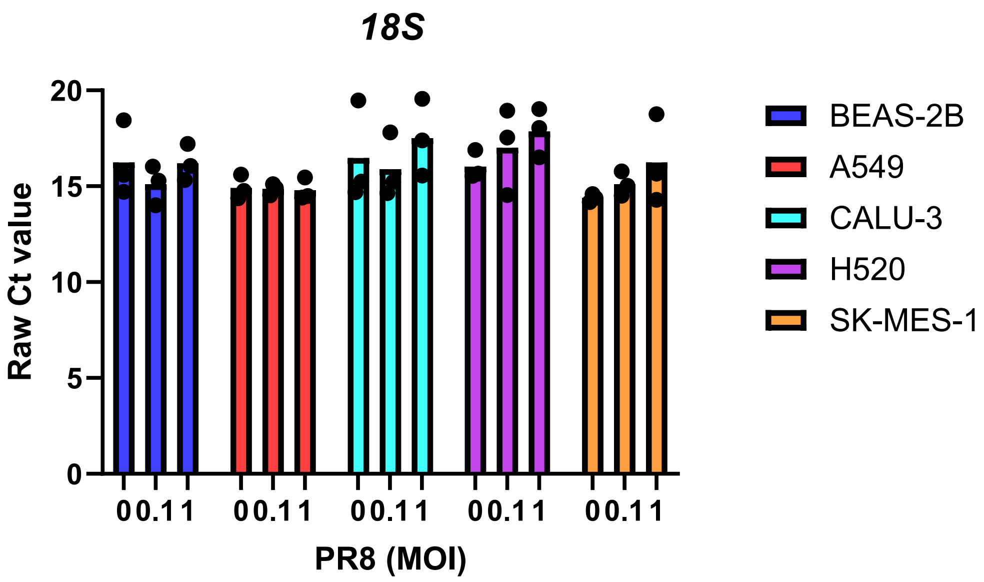

Figure S2. Stability of 18S rRNA expression across cell lines and PR8 infection conditions. Raw 18S Ct values from individual biological replicates are shown for each cell line following infection with PR8 at the indicated MOI.

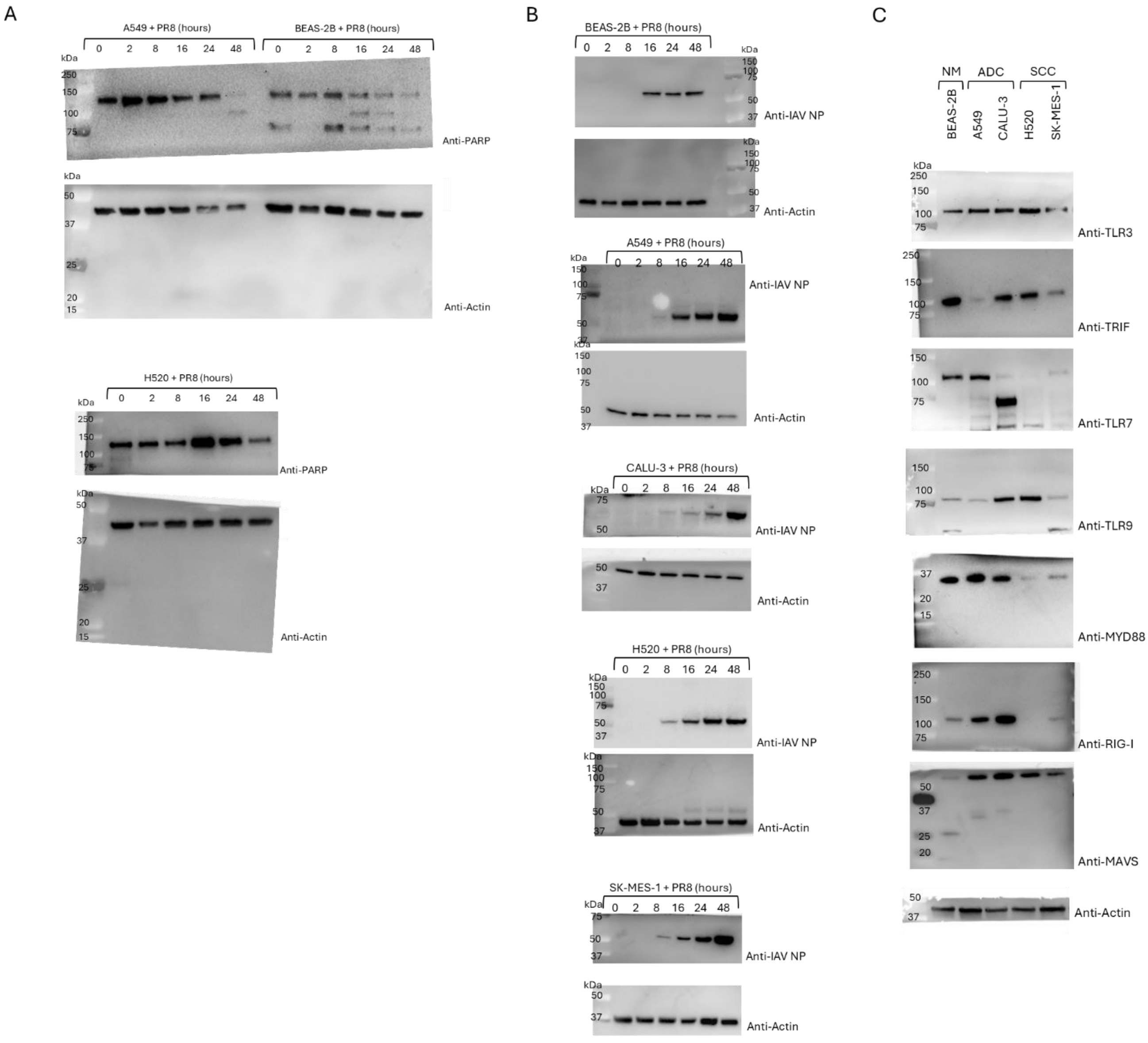

Figure S3. Uncropped blots relevant to (A) Figure 1C, (B) Figure 3B and (C) Figure 5A in the main text.

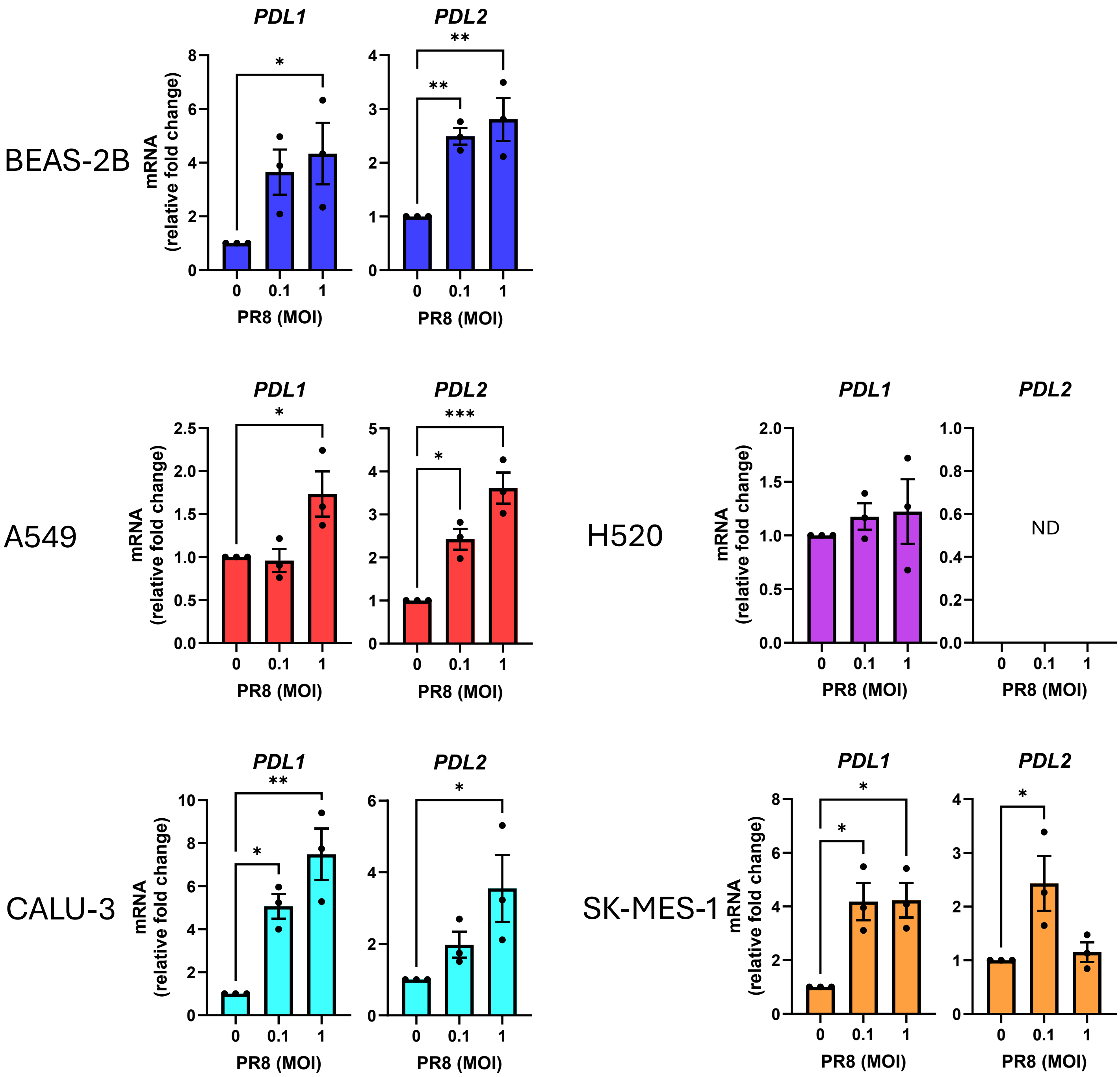

Figure S4. PD-L gene expression analysis in lung epithelial cells following IAV infection. Malignant A549, CALU-3, H520 and SK-MES-1, and non-malignant BEAS-2B lung epithelial cell lines were infected with PR8 virus at MOI of 0, 0.1 and 1 for 24 hours. Gene expression of checkpoint ligands was measured by RT-qPCR. Data is presented relative to 18S housekeeping as a fold-change above uninfected controls ($2^{-\Delta\Delta Ct}$). Genes that failed to reach cycle threshold across the sample group and were below the limit of quantification are denoted as "ND" (not detected). Data are expressed as mean ± SEM from 3 independent biological replicates. Statistical analysis was conducted using a one-way ANOVA test followed by Dunnett's post hoc test for multiple comparisons (*$p < 0.05$, **$p < 0.01$, ***$p<0.001$).
